# Multi-objective exponential distribution optimizer (MOEDO): a novel math-inspired multi-objective algorithm for global optimization and real-world engineering design problems

**DOI:** 10.1038/s41598-024-52083-7

**Published:** 2024-01-20

**Authors:** Kanak Kalita, Janjhyam Venkata Naga Ramesh, Lenka Cepova, Sundaram B. Pandya, Pradeep Jangir, Laith Abualigah

**Affiliations:** 1 Department of Mechanical Engineering, Vel Tech Rangarajan Dr. Sagunthala R&D Institute of Science and Technology, Avadi, 600 062 India; 2 University Centre for Research and Development, Chandigarh University, Mohali, 140413 India; 3 Department of Computer Science and Engineering, Koneru Lakshmaiah Education Foundation, Vaddeswaram, Guntur, 522502 India; 4 Department of Machining, Assembly and Engineering Metrology, Faculty of Mechanical Engineering, VSB-Technical University of Ostrava, 70800 Ostrava, Czech Republic; 5 Department of Electrical Engineering, Shri K.J. Polytechnic, Bharuch, 392 001 India; 6 Department of Biosciences, Saveetha School of Engineering, Saveetha Institute of Medical and Technical Sciences, Chennai, 602 105 India; 7 Computer Science Department, Al al-Bayt University, Mafraq, 25113 Jordan; 8 Hourani Center for Applied Scientific Research, Al-Ahliyya Amman University, Amman, 19328 Jordan; 9 MEU Research Unit, Middle East University, Amman, 11831 Jordan; 10 Department of Electrical and Computer Engineering, Lebanese American University, Byblos, 13-5053 Lebanon; 11 School of Computer Sciences, Universiti Sains Malaysia, 11800 Pulau Pinang, Malaysia; 12 School of Engineering and Technology, Sunway University Malaysia, 27500 Petaling Jaya, Malaysia; 13 Artificial Intelligence and Sensing Technologies (AIST) Research Center, University of Tabuk, 71491 Tabuk, Saudi Arabia

**Keywords:** Mechanical engineering, Computational science

## Abstract

The exponential distribution optimizer (EDO) represents a heuristic approach, capitalizing on exponential distribution theory to identify global solutions for complex optimization challenges. This study extends the EDO's applicability by introducing its multi-objective version, the multi-objective EDO (MOEDO), enhanced with elite non-dominated sorting and crowding distance mechanisms. An information feedback mechanism (IFM) is integrated into MOEDO, aiming to balance exploration and exploitation, thus improving convergence and mitigating the stagnation in local optima, a notable limitation in traditional approaches. Our research demonstrates MOEDO's superiority over renowned algorithms such as MOMPA, NSGA-II, MOAOA, MOEA/D and MOGNDO. This is evident in 72.58% of test scenarios, utilizing performance metrics like GD, IGD, HV, SP, SD and RT across benchmark test collections (DTLZ, ZDT and various constraint problems) and five real-world engineering design challenges. The Wilcoxon Rank Sum Test (WRST) further confirms MOEDO as a competitive multi-objective optimization algorithm, particularly in scenarios where existing methods struggle with balancing diversity and convergence efficiency. MOEDO's robust performance, even in complex real-world applications, underscores its potential as an innovative solution in the optimization domain. The MOEDO source code is available at: https://github.com/kanak02/MOEDO.

## Introduction

Design considerations inherently involve optimization, necessitating the application of suitable optimization techniques and algorithms^1^. Given the intricate nature of contemporary design tasks, conventional optimization strategies rooted in mathematical theories often fall short in delivering timely solutions. For instance, gradient-based algorithms tackle optimization challenges by leveraging the gradient of the target function^2^. Over the past several years, there has been a surge in interest to rectify the shortcomings of classical optimization algorithms (OAs) and introduce more potent OAs^3^. Thanks to technological progress, newer OAs that boast superior efficiency, precision and speed in addressing diverse optimization tasks are gaining traction^4–6^. Moreover, specific challenges like local optima and the irregularities and non-convexities of exploration domains have played a pivotal role in this evolution.

Such constraints on OAs have spurred scholars and industry experts to develop innovative metaheuristic algorithms to navigate diverse optimization hurdles^7,8^. Parallel to the growth of information technology, a plethora of optimization challenges have emerged across sectors like engineering, bioinformatics, operations research and geophysics^9,10^. Numerous optimization issues are categorized as NP-hard, implying their solutions are not achievable within polynomial time unless NP is equivalent to P. As a result, exact mathematical methods are typically reserved for problems of a smaller scale.

Researchers have explored alternative strategies (approximation techniques) to identify feasible solutions within a reasonable timeframe rather than abandoning the effort. These techniques can be broadly categorized into heuristics and metaheuristics. The primary difference between the two is that heuristics are closely tied to the specific nature of a problem, making them effective for particular challenges but less so for others. In contrast, metaheuristics present a more universal algorithmic structure or a black-box optimization tool suitable for almost any optimization problem (OP). These advanced heuristics, designed to address a range of OPs, are termed metaheuristics (MHs). Numerous metaheuristic algorithms (MHAs) have been successfully employed in recent times to navigate complex challenges^11^. A key benefit of these algorithms in addressing intricate optimization tasks is their capability to identify commendable solutions, irrespective of the problem's scale or intricacy^12^. MH algorithms have been utilized across a spectrum of optimization challenges, encompassing both single and multi-objective, as well as continuous and discrete scenarios^13^.

In the real world, problems often present multiple objectives, some of which might conflict with others. As a result, multi-objective optimization problems (MOPs) align more closely with these multifaceted challenges than single-objective optimization does^14^. A common approach to solving MOPs is to treat each objective as an individual OP, addressing them one after the other based on their respective significance^15^. Alternatively, individual solvers can exchange insights across successive iterations.

Techniques for multi-objective optimization can be divided into three primary categories^16,17^: a priori, a posteriori and interactive approaches.

- A priori techniques: these strategies rely on preliminary knowledge about the problem and its objectives. They aim to pinpoint the Pareto optimal solutions even before the optimization process kicks off. A prevalent tactic in these techniques is transforming a MOP into a single-objective challenge at the outset. Notable examples of a priori techniques encompass weighting methods, goal-oriented programming and lexicographic strategies^18^.
- A posteriori technique: these strategies draw from the results secured during the optimization phase. They strive to determine the Pareto optimal solutions post the completion of the optimization. Evolutionary algorithms like genetic algorithms, particle swarm optimization and simulated annealing fall under this category, as do Multi-objective Genetic Algorithms such as NSGA-II, SPEA2, MOEA/D and NSGA-II^19^.
- Interactive techniques: these strategies necessitate human engagement throughout the optimization phase. They grant the decision-maker the ability to engage with the optimization process and offer insights on the outcomes produced by the algorithm^20^. Examples of interactive techniques include interactive genetic algorithms, interactive evolutionary tactics and interactive particle swarm optimization.

**Figure 1:**
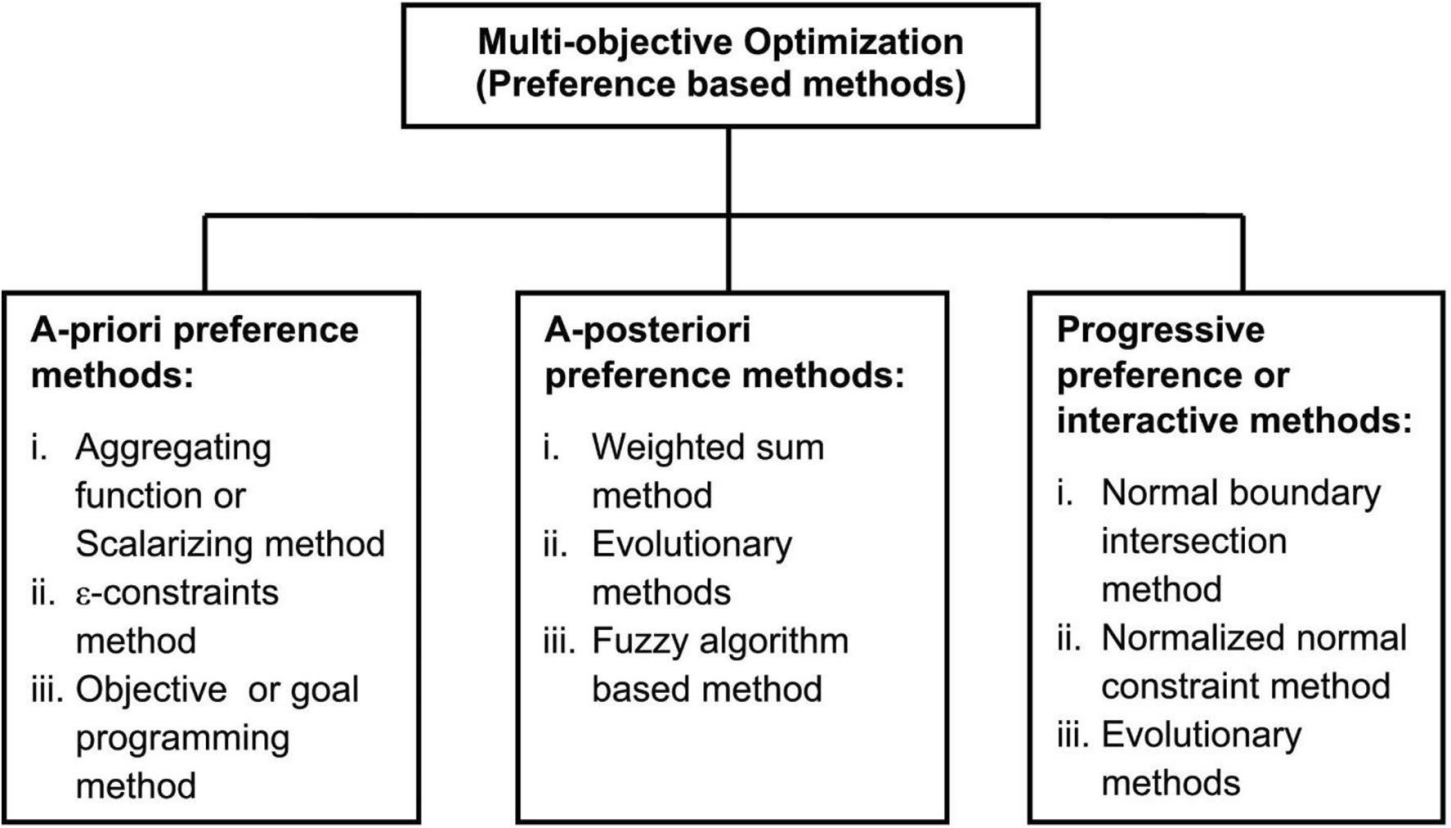
Classification of multi-objective techniques.

While a priori techniques are generally straightforward to deploy and can yield prompt results, they might miss out on capturing the genuine Pareto optimal solutions. On the other hand, a posteriori technique, though potentially offering more precise outcomes, can be intricate to implement and might be resource-intensive. Interactive techniques strike a balance, enabling the decision-maker to influence the outcomes, potentially enhancing solution quality. The techniques are illustrated in Fig. 1.

Multi-objective evolutionary algorithms (MOEAs) have gained popularity as effective a posteriori technique for addressing MOPs. Their strength lies in their ability to approximate the Pareto set (PS) and/or Pareto front (PF) within a single execution^21^. A standard MOEA operates in two main stages for every generation. The first stage employs evolutionary mechanisms like crossover and mutation to generate new solutions in the decision realm. The second stage involves selecting superior solutions from both the newly created and pre-existing solutions for the subsequent generation^22^. Depending on the selection methodologies employed, most contemporary MOEAs can be categorized into four types: domination-based, indicator-based, decomposition-based and hybrid MOEAs^23,24^. To determine the quality of selection, many MOEAs assess the function values of all the freshly produced solutions. Consequently, these algorithms often necessitate numerous function evaluations to achieve convergence^25^. Yet, the long-term outcomes of traditional optimization methods leave much to be desired^26^. Issues such as the sensitivity to the problem's initial estimation, reliance on the precision of the differential equation solver's solution and the risk of becoming ensnared in local optima conventional optimization techniques^27^. In addressing highly nonlinear challenges, there is a heightened risk of settling for a solution that is merely locally optimal^28^.

Metaheuristics for MOPs, commonly referred to as MOEA, can be categorized based on their solution selection methodologies into four primary groups: dominance-based, decomposition-based, indicator-based and hybrid selection mechanisms.

1. Dominance-based MOEAs. These prioritize achieving optimal convergence by assigning fitness scores to individuals according to the Pareto-dominance principle. Additionally, a specific strategy is provided to maintain diversity. A widely recognized approach in this category ranks individuals through rapid non-dominated sorting, emphasizing reduced time complexity^29–32^. When multiple individuals possess the same non-domination rank, density metrics ensure diversity, leading to a more uniform distribution of solutions. However, as the number of objectives increases, the proportion of non-dominated solutions grows exponentially, making Pareto-dominated MOEAs less suitable for many-objective optimization. This can hinder the evolutionary process, diminishing selection pressure, diversity and convergence.
2. Decomposition-based MOEAs. These segment a MOP into multiple scalar optimization sub-tasks using a decomposition strategy. Solutions for each subtask are optimized by conducting evolutionary operations among its various neighboring sub-problems. The most frequently employed decomposition-based MOEA is MOEA/D, introduced by Zhang and Li^30^. The MOEA/D structure can incorporate various traditional single-objective optimization and localized search methods^33–35^.
3. Indicator-based MOEAs. These MOEAs utilize performance metrics related to solution quality as selection standards, guiding the search towards consistent enhancement of the overall population's anticipated attributes^36,37^. IBEA, a pioneering indicator-based MOEA, was developed by Zitzler and Kunzl^38^. It employed a binary indicator to evaluate the comparative benefits of two approximation solution sets, aligning with the decision-maker's preference. Given its compatibility with the Pareto principle, this indicator can be employed to determine fitness. This approach offers a foundational model for future indicator-based MOEAs. Some studies suggest that HV indicators might be a more computationally efficient alternative to other indicators while retaining similar theoretical properties.
4. Hybrid MOEAs. Addressing intricate MOPs often necessitates leveraging the strengths of multiple algorithms, leading to the introduction of various hybrid MO algorithms^39–42^. The primary considerations for hybrid algorithms revolve around the selection of algorithms to merge and the methodology for their integration. Contemporary research predominantly centers on amalgamating different strategies to generate offspring populations. However, there is a need for further exploration in other domains.

Other popular multi-objective (MO) Algorithms include MO ant lion optimizer (MOALO)^43^, MO equilibrium optimizer (MOEO)^44^, MO slime mould algorithm (MOSMA)^45^, MO arithmetic optimization algorithm (MOAOA)^46^, non-dominated sorting ions motion algorithm (NSIMO)^47^, MO marine predator algorithm (MOMPA)^48^, multi-objective multi-verse optimization (MOMVO)^49^, non-dominated sorting grey wolf optimizer (NS-GWO)^50^, MO gradient-based optimizer (MOGBO)^51^, MO plasma generation optimizer (MOPGO)^52^, non-dominated sorting Harris hawks optimization (NSHHO)^53^, MO thermal exchange optimization (MOTEO)^54^, decomposition based multi-objective heat transfer search (MOHTS/D)^55^, Decomposition-Based Multi-Objective Symbiotic Organism Search (MOSOS/D)^56^, MOGNDO Algorithm^57^, Non-dominated sorting moth flame optimizer (NSMFO)^58^, Non-dominated sorting whale optimization algorithm (NSWOA)^59^, Non-Dominated Sorting Dragonfly Algorithm (NSDA)^60^, a reference vector based multiobjective evolutionary algorithm with Q-learning for operator adaptation^61^, a many-objective evolutionary algorithm based on hybrid dynamic decomposition^62^ and use of two penalty values in multiobjective evolutionary algorithm based on decomposition^63^.

Several algorithms have been crafted to adeptly navigate intricate OPs. One notable example is the exponential distribution optimizer (EDO) algorithm introduced by Mohamed Abdel-Basset et al.^64^. This innovative algorithm has been applied across diverse sectors, from engineering challenges to manufacturing processes and scientific modeling. The foundational principle of the EDO algorithm draws integrates two approaches geared towards exploitation and exploration tactics. During the exploitation phase, the algorithm employs three core principles: the absence of memory property, a guiding solution and the exponential disparity observed among the exponential random variables to refine the existing solutions.

In recent times, the exponential distribution optimizer (EDO), a math inspired metaheuristic method rooted in the fractal theorem, was presented. Our research aims to introduce a novel multi-objective iteration of the EDO algorithm tailored for MOPs. The proposed version integrates the NDS, CD and IFM principle and a stochastic learning mechanism. To gauge the potency of this proposed method, we employ distinct benchmark test functions: ZDT^65^, DTLZ^66^, Constraint^67,68^ (CONSTR, TNK, SRN, BNH, OSY and KITA) and real-world engineering design Brushless DC wheel motor^69^ (RWMOP1), Helical spring^68^ (RWMOP2), Two-bar truss^68^ (RWMOP3), Welded beam^70^ (RWMOP4), Disk brake^71^ (RWMOP5). The objective of this assessment is to compare the efficacy of our proposed method against MOMPA, NSGA-II, MOAOA, MOEA/D and MOGNDO, using metrics like generational distance (GD)^34^, inverse generational distance (IGD)^35^, hypervolume^36^, Spacing^37^, Spread^36^ and run time (RT). The approximations of the Pareto-front produced by our method are evaluated using these metrics.

The key contributions of this research are succinctly outlined as follows:

1. Introduction of the multi-objective exponential distribution optimizer (MOEDO) Algorithm, incorporating non-dominated sorting (NDS) and crowding distance (CD) principles.
2. Integration of the information feedback mechanism (IFM) to decompose multi-objective challenges into single-objective sub-tasks, enhancing the algorithm's efficiency.
3. Utilization of the IFM approach to ensure a balanced dynamic between exploration and exploitation, fostering improved convergence and the capability to bypass local minima.
4. Comprehensive evaluation of MOEDO's performance against established multi-objective methods using benchmark datasets like ZDT, DTLZ, CONSTR and real-world engineering design problems.
5. Employment of metrics such as generational distance (GD), inverse generational distance (IGD), hypervolume (HV), spacing, spread and run time (RT) to assess results, demonstrating the superior capabilities of our proposed technique in various test scenarios.

The structure of our comprehensive research is as follows: "Methodology" elaborates on the exponential distribution optimizer. "Results and discussion" provides an in-depth look at our proposed MOEDO method for multi-objective global optimization. "Conclusion" presents our experimental results.

## Methodology

This section delves into the EDO algorithm^64^ fundamental components. We will first discuss the inspiration behind EDO, followed by a detailed explanation of its initialization. We will also examine the explorative and exploitative features of EDO before discussing its mathematical structure.

### Exponential distribution optimizer (EDO)

#### Exponential distribution model

The EDO approach takes its foundational principles from the theories of exponential distribution. This distribution, which is continuous in nature, has been instrumental in explaining several occurrences in the natural world. For instance, the time span between the present moment and the occurrence of an earthquake can be represented using this distribution. Similarly, the time-based likelihood of a car reaching a toll station aligns with the exponential distribution. Exponential random variables have frequently been utilized to shed light on past events, particularly focusing on the duration leading up to a specific incident. Here, we delve into the mathematical construct of the exponential distribution and elucidate its unique attributes. Imagine having an exponential random variable, represented by $x$, associated with a parameter, $\lambda$. This relationship can be expressed as $x\sim {\text{EXP}}(\lambda )$. The Probability Density Function (PDF) of this variable is indicative of the duration by:

1 $$f(x)=\left\{\begin{array}{cc}\lambda {e}^{-\lambda x}& x\ge 0\\ 0,& \, {\text{otherwise}}\end{array}\right.$$

As time is a continuous factor, it must always hold a non-negative value, i.e., $(x\ge 0)$. Significantly, the $\lambda$ parameter, which is always positive, denotes the rate of occurrence in the exponential distribution. The exponential distribution cumulative distribution function (CDF) can be derived through a specific formula:

2 $$F(x)=\left\{\begin{array}{cc}1-{e}^{-\lambda x}& x\ge 0\\ 0,& \, {\text{otherwise}}\end{array}\right.$$

A higher value of the rate of occurrence, $\lambda$, indicates a reduced probability of the concerned random variable.

CDF function ascends, commencing from the foundational exponential rate and intensifying in proportion to the growth of the exponential random variable. For an exponentially distributed random variable, its mean $\left(\mu \right)$ and variance $\left({\sigma }^{2}\right)$ can be articulated using specific mathematical expressions:

3 $$\begin{array}{c}\mu =\frac{1}{\lambda },\\ {\sigma }^{2}=\frac{1}{{\lambda }^{2}}.\end{array}$$

These expressions reveal an inverse relationship between the $\lambda$ parameter and both the mean and variance. To put it succinctly, as $\lambda$ increases, both the mean and variance decline. Moreover, the standard deviation $(\sigma )$ mirrors the mean value and its derivation follows a particular computation

4 $$\sigma =\sqrt{\frac{1}{{\lambda }^{2}}}=\frac{1}{\lambda }=\mu$$

#### The memoryless nature of exponential distribution

One of the unique characteristics of certain statistical probability distributions is the 'memoryless' attribute. This implies that the chance of a forthcoming event transpiring is independent of past events. In simpler terms, previous occurrences do not influence future probabilities. The exponential distribution, which is a continuous type, encapsulates this memoryless attribute, especially when gauging the timespan before an event occurrence. When a random variable, denoted by $x$, adheres to the exponential distribution with this memoryless feature, it means for any positive whole numbers $s$ and $t$ belonging to the series $\left\{\mathrm{0,1},2,\dots ,\infty \right\}$ the following holds true:

5 $$P(x>s+t\mid x\ge s)=P(x>t)\text{.}$$

#### Launching with the initial group

During the onset or the initiation stage, we cultivate a group termed (${X}_{\text{winners}}$), comprising $N$ diversely valued, randomly formed solutions. To depict this search operation, we utilize an assortment of exponential distributions. Every potential solution is perceived as an embodiment of the exponential distribution. The respective positions of each solution are considered as exponential random variables conforming to this distribution. These are then structured as vectors, having a dimension $d$.

6 $${X}_{{{\text{winners}} }_{i}}=\left[{X}_{{{\text{winners}} \, }_{i,1}},{X}_{{{\text{winners}} }_{i,2}},\dots ,X{ \, {\text{winners}} }_{i,d}\right]$$

7 $$X{\text{winners}} \, {s}_{i,j}\in [lb,ub]\text{,} \, i=\mathrm{1,2},\dots ,N\text{,}j=\mathrm{1,2},\dots ,d.$$

In this setup, ${X}_{{\text{winners}} }$ symbolizes the $j$ element of the *i*th candidate within the exponential distribution vector. Following this, we can define the preliminary group, ${X}_{\text{winners}}$.

8 $$\mathrm{Xwinners }=\left[\begin{array}{cccc}{ \, {\text{Xwinner}} }_{\mathrm{1,1}}& { \, {\text{Xwinner}} }_{\mathrm{1,2}}& \dots & { \, {\text{Xwinners}} }_{1,d}\\ { \, {\text{Xwinners}} }_{\mathrm{2,1}}& { \, {\text{Xwinners}} }_{\mathrm{2,2}}& \dots & { \, {\text{Xwinners}} }_{2,d}\\ \vdots & \vdots & \vdots & \vdots \\ { \, {\text{Xwinners}} }_{N,1}& { \, {\text{Xwinner}} }_{N,2}& \dots & { \, {\text{Xwinners}} }_{N,d}\end{array}\right]$$

To randomly generate each variable within the candidate exponential distribution in the solution space, we utilize a particular formula.

9 $$X{ \, {\text{winners}} }_{i,j}=Ib+{\text{rand}}(ub-lb)\text{.}$$

Here, $lb$ and $ub$ delineate the lower and upper limits of the given problem. The term 'rand' signifies a randomly derived number within the span [0,1]. Upon concluding the initiation, we embark on the optimization phase. This leans on the exploratory and refinement skills of our proposed approach over iterative rounds. Subsequent sections will elucidate the two core techniques (exploration and refinement within EDO) for pinpointing the global pinnacle.

#### EDO exploitation strength

The exploitation component of EDO harnesses various facets of the exponential distribution model, including its memoryless trait, exponential rate, typical variance and average value. Additionally, a guiding solution steers the search towards the global peak. Initially in EDO, a set of random solutions is cultivated, mimicking an array of exponential distribution patterns. These solutions undergo evaluation via an objective function and are subsequently ranked in terms of performance. For maximization problems, they are ordered in descending efficiency, while for minimization challenges, they are arranged in ascending order. Areas surrounding a robust solution are fertile grounds for pinpointing the global apex. This is why several algorithms probe spaces around robust solutions, drawing weaker ones towards them. Therefore, the global apex quest focuses on the guiding solution. The guiding solution, labeled ($Xguide_{{\text{~}}}^{{{\text{time}}}}$), is derived from the mean of the top three solutions in a sorted group.

10 $$Xguide^{{{\text{time~}}}} = \frac{{{\text{Xwinners~~}}_{{{\text{best~1}}}}^{{{\text{time~}}}} + {\text{~Xwinners~~}}_{{{\text{best~}}2}}^{{{\text{time~}}}} + {\text{~Xwinners~}}_{{{\text{best~}}3}}^{{{\text{time~}}}} }}{3}$$

In this context, time replaces the term iteration, alluding to the period until the subsequent event in the exponential distribution. The maximum number of iterations is symbolized by Max_time. This guiding solution offers invaluable insights about the global apex. Instead of exclusively leaning on the current best solution, the guiding one is prioritized. Though the leading solution has vital details about the global peak, an exclusive focus on it could lead to convergence around a local maximum. By introducing a current guiding solution, this challenge is mitigated. To emulate the memoryless characteristic of the exponential distribution, a matrix, termed memoryless, is formulated. This matrix houses the latest solutions, irrespective of their present efficiency scores. Initially, it mirrors the original population. Following this, solutions generated at the present time, regardless of their efficiency, are stored in the matrix, dismissing their past contributions. Operating in line with the memoryless property, if current solutions do not fare well against their counterparts in the original group, their success probabilities in subsequent iterations match those of the present. Therefore, past failures do not dictate future outcomes. Within the memoryless matrix, there are two categories of solutions: winners and losers. While losers can still contribute to the optimization process alongside winners, a solution is deemed victorious if its efficiency surpasses that of its counterpart in the ${X}_{{\text{winners}} }$ group. If a solution is updated in both the ${X}_{{\text{winners}} }$ and memoryless matrices, it a winner, otherwise, it a loser.

Our designed exploitation model, focused on updating solutions adhering to the exponential distribution, relies on both winning and losing solutions. The updated solution, $\left({V}_{i}^{timx+1}\right)$ is modelled around a specific involving random and adaptive parameters. This strives to locate the global peak near an efficient solution, creating a new solution for future populations. The delves deep, examining how winners and losers navigate within the search space, leveraging valuable data from winners:

11 $${V}_{i}^{{{time}} \, +1}=\left\{\begin{array}{l} \, \text{a.} \, \left({ \, {\text{memoryless}} }_{i}^{{{time}} }-{\sigma }^{2}\right)+{ \, \text{b.Xguide} }^{{{time}} } \, {\text{if}} \, {{\text{Xwinners}} }_{i}^{{{time}} }\text{=}{ \, {\text{memoryless}} }_{i}^{{{time}} } \, \\ \, \text{b.} \, \left({ \, {\text{memoryless}} }_{i}^{{{time}} }-{\sigma }^{2}\right)+{\text{log}}\left(\phi \right).{{\text{Xwinners}} }_{i}^{{{time}} }, Otherwise\\ \end{array}\right.$$

12 $$a=(f{)}^{10},b=(f{)}^{5}, f=2\times \, {\text{rand}} \, -1$$

Additionally, the exponential rate, in relation to the mean, can be deduced:

13 $$\lambda =\frac{1}{\mu },$$

14 $$\mu =({ \, {\text{memoryless}} }_{i}^{{{time}} }+{ \, {\text{Xguide}} }^{{{time}} })/2$$

The exponential mean arises from averaging the guiding solution and the associated memoryless vector, which can be either a winner or loser.

#### EDO exploration potential

This section delves into the exploration aspect of the introduced algorithm. The exploration segment pinpoints potential areas within the search domain that likely house the ideal, global solution. The EDO exploratory blueprint derives from two prominent solutions, or winners, from the primary population, which adhere to the exponential distribution pattern:

15 $${V}_{i}^{{{time}} \, +1}={{\text{Xwinners}} }_{i}^{{{time}} \, }-{M}^{{{time}} \, }+\left(c{Z}_{1}+(1-c){Z}_{2}\right)$$

16 $${M}^{{{time}} \, }=\frac{1}{N}\cdot \sum_{j=1}^{N} {\text{Xwinner}}{s}_{j,i}^{{{time}} \, }, j=\mathrm{1,2},\dots ..,d$$

Updating the new solution involves with ${M}^{{time}}$ representing the average of all solutions sourced from the primary group. This average is computed by adding all exponential random variables from the same dimension and then dividing by the total population, denoted as $N$. The term $c$ signifies a refined parameter, indicating the proportion of information drawn from the ${Z}_{1}$ and ${Z}_{2}$ vectors towards the contemporary solution and is formulated as:

17 $$c=d\times f,d=\frac{1- \, {{time}} \, }{ \, \text{Max\_time}}$$

Here, $d$ stands as a flexible parameter. Initially set to zero, it undergoes gradual decrement as time progresses. Here, 'time' alludes to the present moment, while 'Max_time' signifies the comprehensive duration or iterations. Both ${Z}_{1}$ and ${Z}_{2}$ are viewed as potential vectors, formed by:

18 $$Z_{1} = M - D_{1} + D_{2} ,Z_{2} = M - D_{2} + D_{1} ,D_{1} = M - {\text{Xwinners}}_{{rand1}} ,D_{2} = M - {\text{Xwinners}}_{{rand2}}$$

Furthermore, ${D}_{1}$ and ${D}_{2}$ delineate the distance between the average solution and the 'winners' randomly picked from the initial population. At the inception of the optimization journey, a notable disparity exists between the average solution and the standout performers. Yet, as the process nears its end, this gap between the prominent solutions and their corresponding variances narrows. To establish the ${Z}_{1}$ and ${Z}_{2}$ vectors, exploration is conducted around the average solution, aided by a pair of randomly chosen outstanding solutions.

#### Optimizing with exponential distribution optimizer (EDO)

The EDO method we are introducing follows a series of steps to thoroughly navigate the search space, aiming for the global optimum. Initially, we create a collection of solutions, randomly generated and marked by a wide range of values. The search process is depicted using various exponential distributions and as such, the location of each solution can be seen as random variables adhering to this distribution. We design a matrix without memory to mimic the absence of memory and initially, it mirrors the original group of solutions. Leveraging the exploratory and refining stages of our method, every solution starts moving closer to the global optimum over time. During the refining stage, the matrix without memory is used to store the outcomes from the prior step, irrespective of their past, allowing them to play a pivotal role in shaping the new solutions. This leads to the categorization of solutions into two groups: the successful ones and the unsuccessful ones. Additionally, we incorporate various properties of the exponential distribution, like its mean, rate and variance. The successful solution is guided by a leading solution, while the unsuccessful one follows the successful one, aiming to discover the global optimum nearby. In the exploration stage, the fresh solution is influenced by two randomly chosen successful solutions from the initial group and the average solution. At the start, both the average solution and its variance are distant from the global optimum. However, the gap between the average solution and the global optimum narrows down until it reaches its lowest point during the optimization. A toggle parameter determines whether to embark on the exploration or refining stage, based on a probability where $(a<0.5)$.

19 $${V}_{i}^{{{time}} \, +1}=\left\{\begin{array}{l} \, \text{a.} \, \left({ \, {\text{memoryless}} \, }_{i}^{{{time}} \, }-{\sigma }^{2}\right)+{ \, \text{b.Xguide} \, }^{{{time}} \, } \, {\text{if}} \, {{\text{Xwinners}} \, }_{i}^{{{time}} \, }\text{=}{ \, {\text{memoryless}} \, }_{i}^{{{time}} \, } \, \\ \, \text{b.} \, \left({ \, {\text{memoryless}} \, }_{i}^{{{time}} \, }-{\sigma }^{2}\right)+{\text{log}}\left(\phi \right).{{\text{Xwinners}} \, }_{i}^{{{time}} \, }, Otherwise if (a<0.5)\\ {{\text{Xwinners}} \, }_{i}^{{{time}} \, }-{M}^{{{time}} \, }+\left(c{Z}_{1}+\left(1-c\right){Z}_{2}\right), Otherwise\end{array}\right.$$

After crafting the new solutions, each solution boundaries are verified and then they are stored in the matrix without memory. A selection strategy is employed to incorporate the new solutions from both stages into the initial group. If a new solution proves beneficial, it integrated into the primary group. By the optimization conclusion, all solutions cluster around the global optimum. In the best solution, both the mean and variance are anticipated to be minimal, while the scale parameter λ is expected to be significant. The pseudo code of single objective EDO shown in Algorithm 1.

**Algorithm 1:**
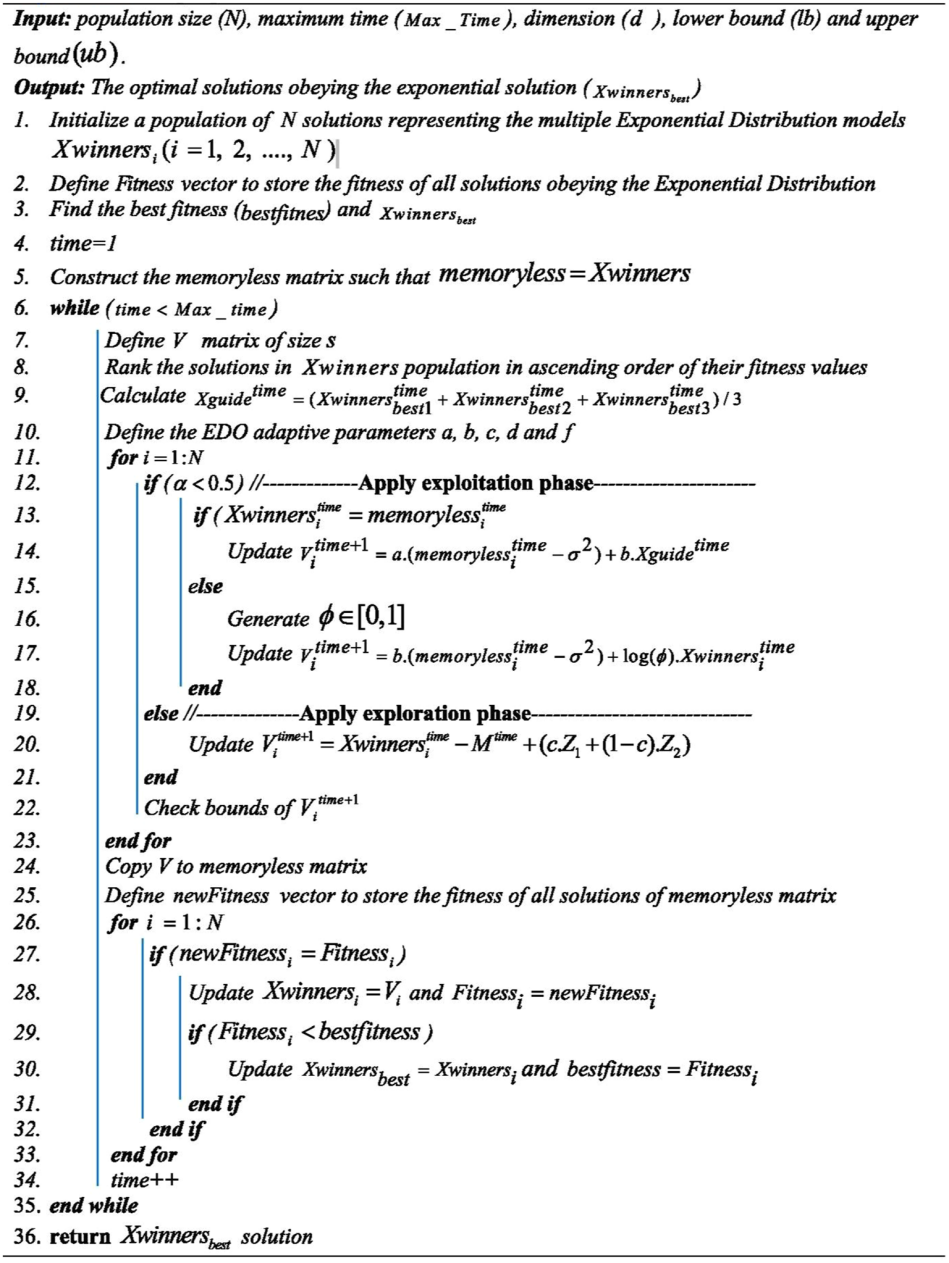
The propsed EDO.

### Proposed multi-objective exponential distribution optimizer (MOEDO)

#### Preliminaries of multi-objective optimization

**Figure 2:**
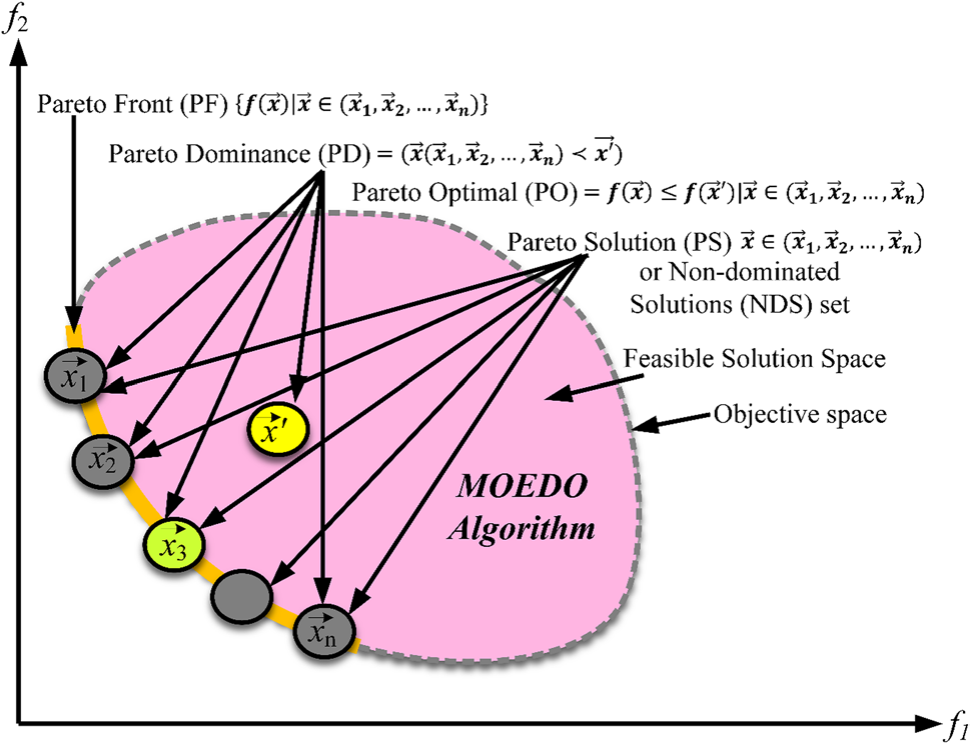
Multi-objective all definitions in search space of MO-problem.

In multi-objective optimization tasks (MOPs), there is a simultaneous effort to minimize or maximize at least two clashing objective functions. While a single-objective optimization effort zeroes in on one optimal solution with the prime objective function value, MOO presents a spectrum of optimal outcomes known as Pareto optimal solutions. An elaboration on the idea of domination and associated terminologies are illustrated in Fig. 2.

#### Multi-objective exponential distribution optimizer (MOEDO)

**Figure 3:**
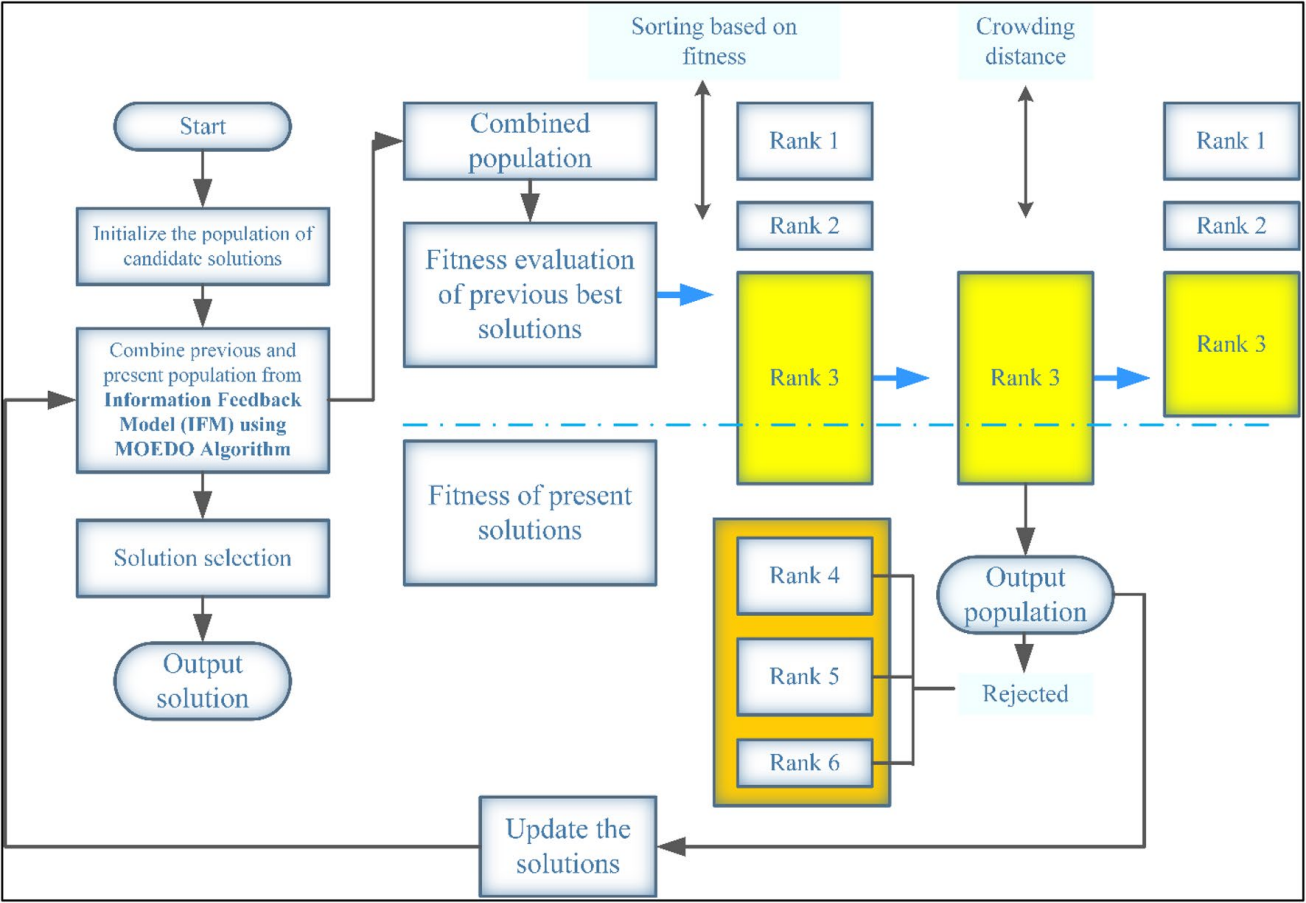
The procedure of the NDS approach based on MOEDO algorithm.

**Figure 4:**
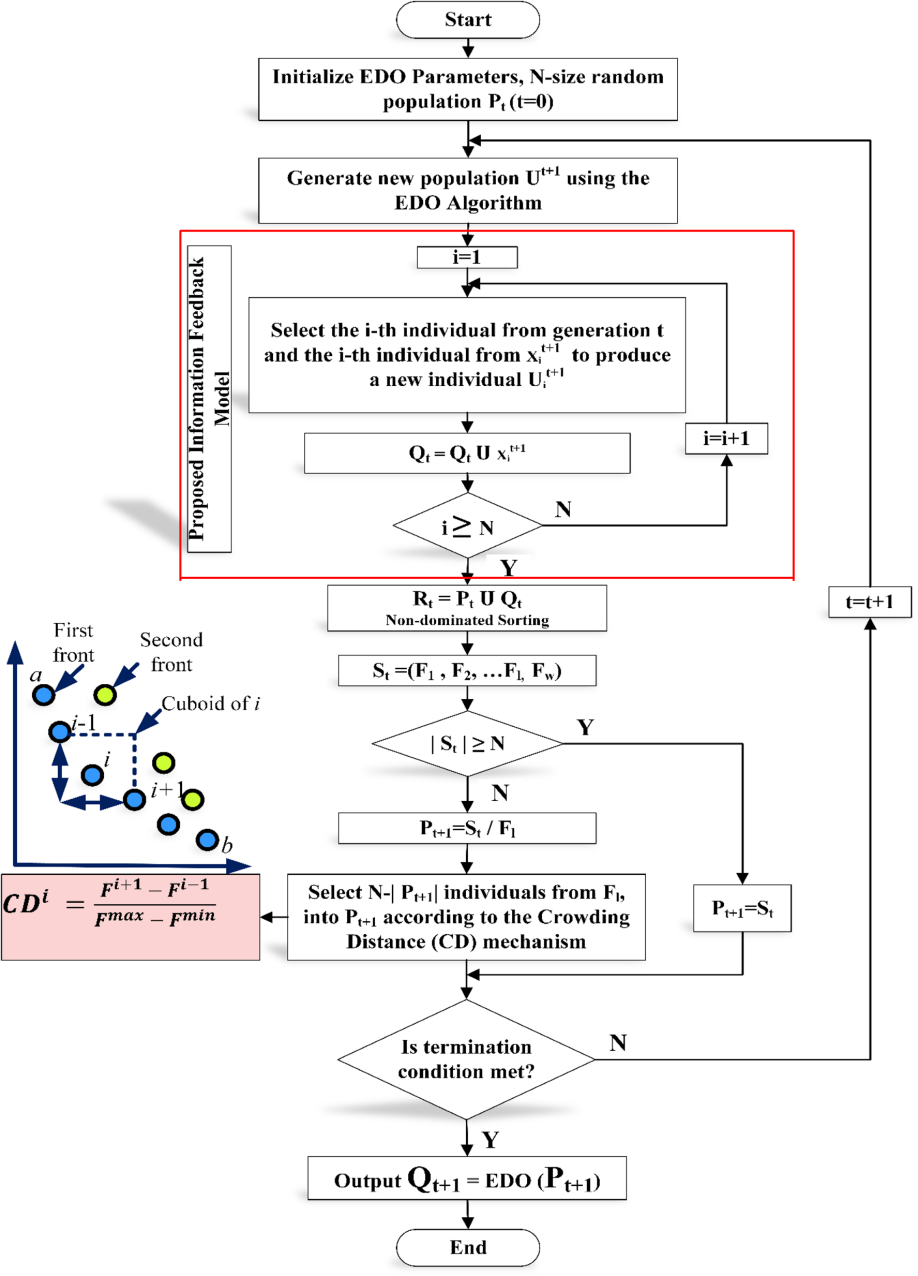
Flowchart of MOEDO algorithm.

MOEDO algorithm starts with a random population of size $N$. the current generation is $t, {x}_{i}^{t}$ and ${x}_{i}^{t+1}$ the $i$th individual at $t$ and $(t+1)$ generation. ${u}_{i}^{t+1}$ the $i$th individual at the $(t+1)$ generation generated through the EDO algorithm and parent population ${P}_{t}$. the fitness value of ${u}_{i}^{t+1}$ is ${f}_{i}^{t+1}$ and ${U}^{t+1}$ is the set of ${u}_{i}^{t+1}$. Then, we can calculate ${x}_{i}^{t+1}$ according to ${u}_{i}^{t+1}$ generated through the EDO algorithm and Information Feedback Mechanism (IFM)^72^ Eq. (20).

20 $${{x}_{i}^{t+1}={\partial }_{1}{u}_{i}^{t+1}+{\partial }_{2}{x}_{k}^{t}; {\partial }_{1}=\frac{{f}_{k}^{t}}{{f}_{i}^{t+1}+{f}_{k}^{t}}, {\partial }_{2}=\frac{{f}_{i}^{t+1}}{{f}_{i}^{t+1}+{f}_{k}^{t}}{\partial }_{1}+{\partial }_{2}=1}$$

where ${x}_{k}^{t}$ is the $k$ th individual we chose from the $t$ th generation, the fitness value of ${x}_{k}^{t}$ is ${f}_{k}^{t},{\partial }_{1}$ and ${\partial }_{2}$ are weight coefficients. Generate offspring population ${Q}_{t}$. ${Q}_{t}$ is the set of ${x}_{i}^{t+1}.$ The combined population ${R}_{t}={P}_{t}\cup {Q}_{t}$ is sorted into different *w*-non-dominant levels $\left({F}_{1},{F}_{2},\dots ,{F}_{l}\dots ,{F}_{w}\right)$. Begin from ${F}_{1}$, all individuals in level 1 to $l$ are added to ${S}_{t}={\bigcup }_{i=1}^{l} {F}_{i}$ and remaining members of ${R}_{t}$ are rejected illustrated in Fig. 3. If $\left|{S}_{t}\right|=N$ no other actions are required and the next generation is begun with ${P}_{t+1}={S}_{t}$ directly. Otherwise, solutions in ${S}_{t}/{F}_{l}$ are included in ${P}_{t+1}$ and the remaining solutions $N-{\sum }_{i=0}^{l-1} \left|{F}_{i}\right|$ are selected from ${F}_{l}$ according to the crowding distance (CD) mechanism, the way to select solutions is according to the CD of solutions in ${F}_{l}$. The larger the crowding distance, the higher the probability of selection and check termination condition is met. If the termination condition is not satisfied, $t=t+1$ than repeat and if it is satisfied, ${P}_{t+1}$ is generated represent in Algorithm 2, it is then applied to generate a new population ${Q}_{t+1}$ by EDO algorithm. Such a careful selection strategy is found to computational complexity of $M$-Objectives $O\left({N}^{2}M\right)$. MOEDO that incorporates proposed information feedback mechanism to effectively guide the search process, ensuring a balance between exploration and exploitation. This leads to improved convergence, coverage and diversity preservation, which are crucial aspects of multi-objective optimization. MOEDO algorithm does not require to set any new parameter other than the usual EDO parameters such as the population size, termination parameter and their associated parameters. The flow chart of MOEDO algorithm can be shown in Fig. 4**.**

**Algorithm 2:**
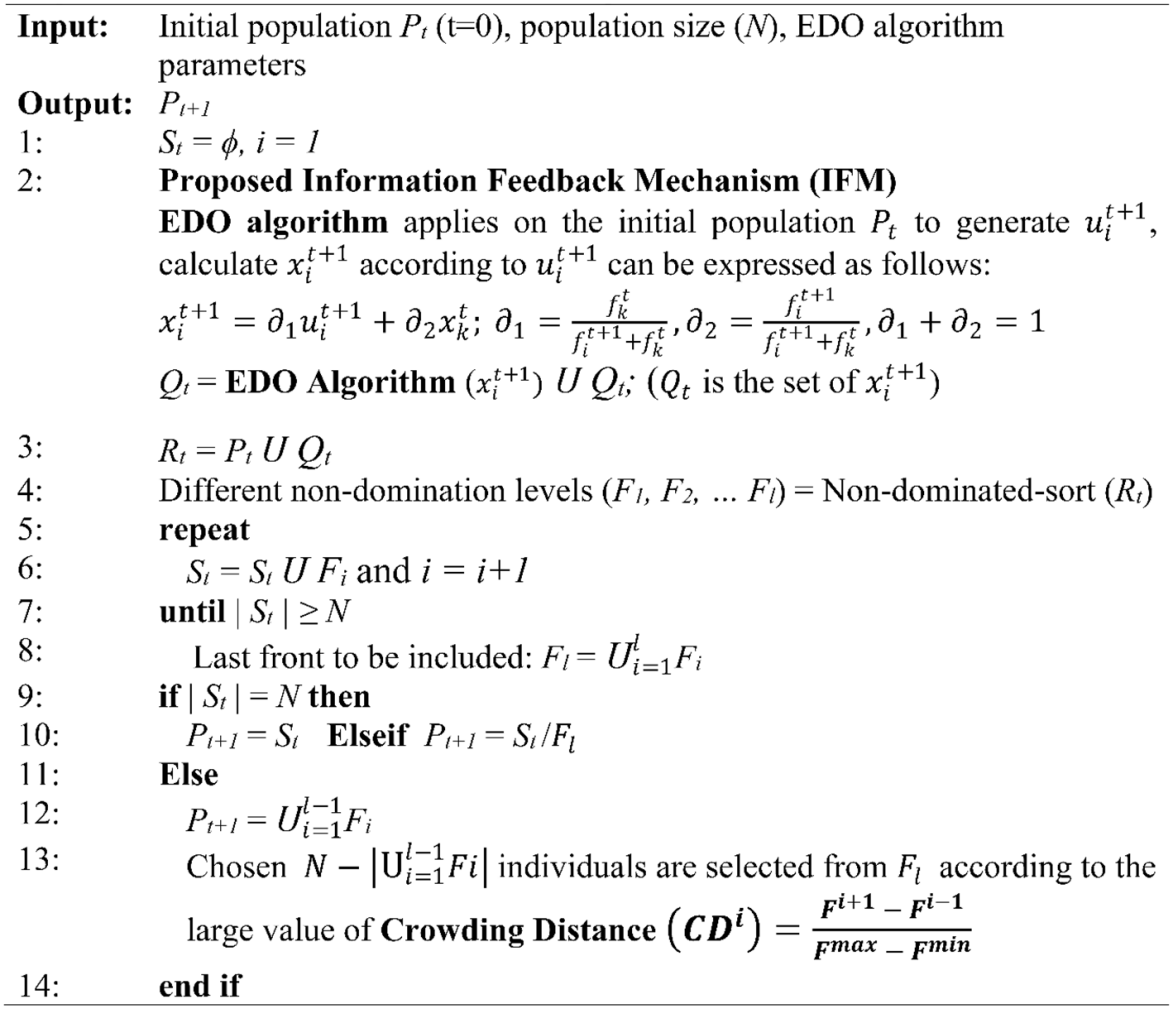
Generation of *t* of MOEDO Algorithm with IFM procedure

## Results and discussion

In this section, we present the results derived from our research and experiments, which were conducted to evaluate the efficacy of the proposed method and showcase its capabilities. To ensure robust and statistically significant results, each experiment was replicated 30 times independently with population size = 40 and maximum number of iterations = 500. All techniques were executed using MATLAB R2020a on an Intel Core i7 computer equipped with a 1.80 GHz processor and 8 GB RAM.

### Description of benchmark test functions

Our experimental evaluation of the MOEDO algorithm's performance utilized three separate benchmark test set functions:

- Zitzler–Deb–Thiele (ZDT) test suite: from this benchmark collection^31^, we selected ZDT1, ZDT2, ZDT3, ZDT4, ZDT 5 and ZDT6 (Appendix A). A brief overview of these problems is as follows: ZDT1 presents a continuous and uniformly distributed convex Pareto front. ZDT2 showcases a concave Pareto front. ZDT3 displays five non-convex discontinuous fronts. ZDT4 has multiple local Pareto Fronts and ZDT6 features a disjointed Pareto front with an irregular mapping between the objective function space and decision variable space. Each ZDT function typically encompasses two objective functions, a common feature in Pareto optimization, especially in engineering contexts.
- Deb–Thiele–Laumanns–Zitzler (DTLZ) test suite: the DTLZ (Appendix B and Appendix C) suite, crafted by Deb et al.^32^, stands out from other multi-objective test challenges due to its adaptability to any objective count. This unique feature has facilitated numerous recent studies into what are commonly termed as many objective challenges. The DTLZ suite encompasses nine test functions, but only DTLZ8 and DTLZ9 have side constraints.
- Constraint^67,68^ CONSTR, TNK, SRN, BNH, OSY and KITA (Appendix D) and real-world engineering design (Appendix E) Brushless DC wheel motor^69^ (RWMOP1), Helical spring^68^ (RWMOP2), Two-bar truss^68^ (RWMOP3), Welded beam^70^ (RWMOP4), Disk brake^71^ (RWMOP5).

### Performance metrics for evaluation

**Figure 5:**
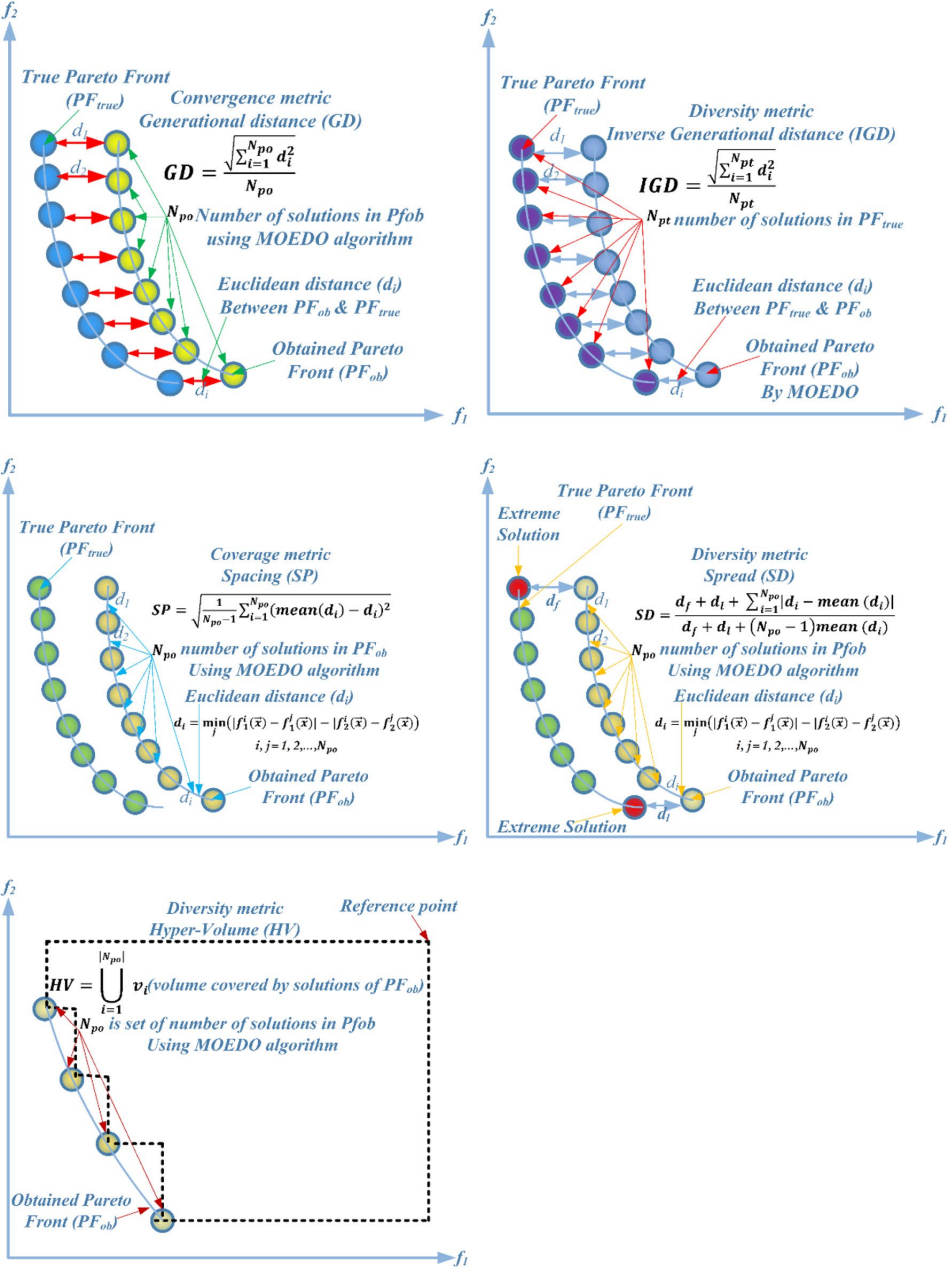
Mathematical and schematic view of the GD, IGD, SP, SD and HV metrics.

For this research, we employed six performance metrics: hypervolume (HV), generational distance (GD), inverted generational distance (IGD), spread (SD), spacing (SP) and run time (RT). Subsequently, we provide a concise explanation of each metric to enhance comprehension shown in Fig. 5. a statistical evaluation. “+/−/~” Wilcoxon signed-rank test (WSRT) was conducted at a significance level of 0.05 between the total amount of test problems on which the corresponding optimizers has a better performance, a worse performance and an equal performance for solving MO problems.

### Performance analysis on benchmark test functions

#### Analysis using the GD metric

Table 1 showcases the final solution distributions when juxtaposing our MOEDO algorithm against MOMPA, NSGA-II, MOAOA, MOEA/D and MOGNDO using the GD metric. Within the ZDT benchmark, our algorithm emerged as the top performer, particularly excelling in ZDT1, ZDT2, ZDT3 and ZDT6 in terms of mean and standard deviation. In the DTLZ benchmark, MOEDO was showed commendable performance in 9/14 problems. For the constraint test suite, the third benchmark, MOEDO outshined in both the best and average metrics, especially in SRN, OSY and KITA. A WRST test was conducted to determine the overall standing of each multi-objective method concerning the GD metric across all benchmarks. The results, presented in Table 1's, place MOEDO at the top and MOMPA bottom behind other algorithms across all benchmarks.

**Table 1:** Results of GD metric of different multi-objective algorithms on ZDT, DTLZ and constraint benchmark problems.

Problem	M	MOEDO	MOMPA	NSGA-II	MOAOA	MOEA/D	MOGNDO
ZDT1	2	3.5899e−5 (1.66e−5) +	9.6778e−3 (2.99e−3) −	8.2443e−4 (3.28e−4) −	2.9289e−4 (7.01e−5) =	1.0291e−3 (3.16e−4) −	3.1913e−4 (1.39e−4)
ZDT2	2	5.5879e−5 (1.90e−5) +	6.8841e−3 (3.43e−3) −	5.5661e−4 (2.37e−4) −	1.7784e−4 (4.77e−5) =	9.6730e−4 (7.50e−4) =	2.4053e−4 (1.12e−4)
ZDT3	2	3.1051e−5 (8.12e−6) +	1.7320e−2 (9.64e−3) −	4.1266e−4 (1.23e−4) −	1.2555e−4 (4.22e−5) +	4.3901e−3 (3.51e−3) −	2.0301e−4 (6.96e−5)
ZDT4	2	4.9930e−4 (2.42e−4) =	1.3619e−2 (1.86e−2) −	1.0777e−3 (7.58e−4) =	4.4432e−4 (1.99e−4) =	8.4438e−3 (7.41e−3) −	6.3176e−4 (2.81e−4)
ZDT5	2	1.7608e−1 (6.11e−2) −	3.8417e−1 (8.95e−2) −	1.0727e−1 (4.11e−2) =	8.5044e−2 (3.68e−2) =	1.8600e−1 (1.08e−2) −	9.5113e−2 (2.25e−2)
ZDT6	2	4.3546e−5 (2.85e−5) +	1.3273e−2 (4.57e−3) −	7.6474e−4 (4.63e−4) −	2.6681e−4 (1.67e−4) =	1.5860e−3 (5.35e−4) −	3.3853e−4 (2.43e−4)
DTLZ1	2	6.0144e−4 (8.85e−4) +	1.6082e−3 (9.63e−4) +	1.8068e−4 (1.32e−4) +	2.1494e−4 (1.91e−4) +	2.9252e−4 (1.65e−4) +	1.0717e + 1 (1.13e + 0)
	3	5.1596e−4 (2.15e−4) +	2.0353e−3 (1.89e−3) +	1.6209e−3 (2.44e−3) +	7.6726e−4 (3.35e−4) +	4.0226e−4 (1.71e−4) +	7.1523e + 0 (1.34e + 0)
DTLZ2	2	6.5895e−6 (6.96e−7) +	2.9149e−4 (1.13e−4) +	8.2707e−6 (2.05e−6) +	6.8522e−5 (2.62e−5) +	1.1447e−4 (2.90e−5) +	1.1704e−1 (1.32e−2)
	3	5.9406e−4 (2.19e−5) +	5.4216e−4 (3.69e−5) +	5.1050e−4 (4.98e−6) +	9.7976e−4 (1.18e−4) +	1.1220e−3 (7.88e−5) +	7.8219e−2 (5.65e−3)
DTLZ3	2	3.7702e−1 (5.19e−1) +	1.4681e + 0 (9.55e−1) +	4.2170e−1 (2.97e−1) +	1.0600e−1 (1.44e−1) +	8.5279e−2 (9.96e−2) +	7.4791e + 1 (9.01e + 0)
	3	2.4250e−1 (2.75e−1) +	9.0255e−1 (4.03e−1) +	3.6571e−1 (1.80e−1) +	4.2306e−1 (4.40e−1) +	2.5751e−1 (4.41e−1) +	4.1806e + 1 (4.97e + 0)
DTLZ4	2	5.5527e−6 (2.97e−6) +	1.8542e−4 (4.80e−5) +	8.5496e−6 (1.56e−6) +	4.3208e−5 (3.94e−5) +	1.1022e−4 (2.54e−5) +	1.6174e−1 (1.32e−2)
	3	5.7792e−4 (1.68e−5) +	5.2973e−4 (1.91e−5) +	5.1197e−4 (9.47e−6) +	9.7575e−4 (4.24e−4) +	1.2343e−3 (1.29e−4) +	1.4498e−1 (3.38e−2)
DTLZ5	2	7.4741e−6 (3.05e−6) +	1.7263e−4 (7.41e−5) +	8.1144e−6 (1.22e−6) +	6.4584e−5 (2.59e−5) +	1.0878e−4 (5.69e−5) +	1.2087e−1 (6.70e−3)
	3	9.3663e−6 (8.02e−6) +	4.2770e−2 (1.85e−2) +	2.2465e−4 (6.12e−5) +	2.6687e−4 (7.29e−5) +	2.9505e−4 (1.45e−4) +	1.0173e−1 (4.82e−3)
DTLZ6	2	4.3256e−6 (2.40e−7) +	4.6240e−6 (4.15e−7) +	4.4398e−6 (2.44e−7) +	4.9037e−6 (2.61e−7) +	4.8632e−6 (1.36e−7) +	5.6426e−6 (4.68e−7)
	3	4.4876e−6 (2.71e−7) +	8.9144e−2 (4.85e−2) −	4.6638e−6 (3.65e−7) +	4.6423e−6 (1.39e−7) +	4.8205e−6 (2.03e−7) +	9.1963e−6 (1.56e−6)
DTLZ7	2	8.6023e−6 (4.18e−7) +	5.2207e−3 (1.65e−3) −	6.0504e−5 (1.49e−5) =	4.5904e−5 (1.77e−5) =	9.6324e−5 (3.61e−5) =	6.6069e−5 (4.57e−5)
	3	8.7151e−4 (6.20e−5) +	8.7116e−3 (5.66e−4) +	2.5299e−3 (3.50e−4) +	2.4906e−3 (3.02e−4) +	2.8349e−3 (3.25e−4) +	5.2801e−2 (4.03e−2)
CONSTR	2	3.3253e−4 (1.51e−5) −	5.0625e−3 (7.73e−4) −	3.5477e−4 (2.75e−5) −	7.0308e−4 (1.08e−3) −	1.6442e−4 (3.92e−5) =	1.2320e−4 (1.08e−5)
TNK	2	1.6889e−4 (4.67e−5)	4.8323e−3 (9.57e−4)	1.3526e−4 (1.44e−5)	1.1308e−2 (2.66e−3) =	2.4982e−2 (4.13e−3) =	1.1896e−2 (1.37e−3)
SRN	2	2.5602e−2 (1.46e−2)	1.9313e + 0 (3.27e−1)	4.9892e−2 (4.19e−3)	7.1254e−3 (4.86e−3) =	1.3429e + 0 (5.35e−1)	2.4982e−2 (4.13e−3) =
OSY	2	7.6376e−1 (7.21e−2)	7.4381e + 0 (7.75e + 0)	8.2825e−1 (1.66e−1)	4.5599e−3 (8.54e−4)	3.4905e + 0 (2.88e + 0)	6.0564e−3 (1.06e−3) =
BNH	2	4.9634e−3 (3.25e−4) +	2.3654e−2 (3.55e−3) −	1.1613e−2 (2.15e−3) =	9.1847e−3 (1.38e−3) +	4.8497e−3 (4.19e−4) +	1.1839e−2 (1.26e−3)
KITA	2	1.1858e−3 (1.06e−5) +	5.7029e−3 (1.52e−3) −	1.2696e−3 (4.80e−5) =	1.4396e−3 (6.63e−5) −	1.4160e−3 (6.89e−5) −	1.2490e−3 (3.76e−5)

#### Analysis using the IGD metric

Table 2 presents the final solution distributions when comparing MOEDO algorithm against MOMPA, NSGA-II, MOAOA, MOEA/D and MOGNDO using the IGD metric. For the ZDT3 and ZDT 6 test function, our algorithm surpassed its counterparts. In the DTLZ benchmark, MOEDO was notably superior in 8/14 metrics. Furthermore, in the third benchmark, MOEDO exhibited superior performance, especially in TNK, OSY and KITA both in the best and average metrics. MOMPA and MOEA/D consistently underperformed in all benchmarks. The WRST test's results, displayed in Table 2's final row, highlight MOEDO's dominance, securing the top rank among the evaluated multi-objective variants.

**Table 2:** Results of IGD metric of different multi-objective algorithms on ZDT, DTLZ and constraint benchmark problems.

Problem	M	MOEDO	MOMPA	NSGA-II	MOAOA	MOEA/D	MOGNDO
ZDT1	2	5.7530e−3 (2.65e−3) +	1.5182e−1 (8.75e−2) −	4.1114e−2 (4.50e−2) =	7.5748e−3 (3.25e−3) +	3.2328e−2 (2.39e−2) =	3.1042e−2 (3.23e−2)
ZDT2	2	2.9093e−2 (4.63e−2) +	1.9531e−1 (9.54e−2) =	1.9194e−1 (6.85e−2) −	4.1784e−2 (2.97e−2) =	1.6212e−1 (1.77e−1) =	1.1055e−1 (8.26e−2)
ZDT3	2	8.3171e−2 (1.01e−1) =	1.4706e−1 (6.10e−2) =	3.1223e−2 (3.25e−2) =	1.4433e−1 (5.96e−2) −	4.9961e−2 (3.27e−2) =	8.3405e−2 (7.58e−2)
ZDT4	2	6.8189e−3 (1.51e−3) +	1.8163e−1 (1.27e−1) −	5.0940e−2 (5.00e−2) =	2.0976e−1 (1.39e−1) −	7.8724e−2 (5.36e−2) −	3.2366e−2 (3.31e−2)
ZDT5	2	5.3770e−1 (1.36e−1) =	2.6373e + 0 (7.14e−1) −	5.9685e−1 (1.53e−1) =	1.7015e + 0 (4.09e−1) −	7.9240e + 0 (2.90e−1) −	5.8823e−1 (1.02e−1)
ZDT6	2	4.7754e−3 (1.11e−3) =	7.2220e−2 (2.16e−2) −	8.4752e−3 (3.96e−3) −	5.1878e−3 (9.39e−5) =	1.2589e−2 (3.87e−3) −	4.5874e−3 (1.22e−3)
DTLZ1	2	2.7364e−3 (1.00e−3) +	1.5813e−2 (1.12e−2) +	3.8205e−3 (1.18e−3) +	3.0759e−3 (1.49e−3) +	7.3334e−2 (9.73e−3) +	2.2978e + 1 (3.67e + 0)
	3	2.4037e−2 (5.07e−3) +	6.8935e−2 (5.51e−2) +	2.7682e−2 (8.31e−4) +	2.2190e−2 (1.53e−3) +	1.9307e−1 (3.21e−2) +	2.4965e + 1 (3.82e + 0)
DTLZ2	2	3.9800e−3 (1.76e−5) +	5.4992e−3 (1.13e−3) +	5.0270e−3 (2.60e−4) +	4.1957e−3 (7.05e−5) +	1.5957e−2 (1.41e−3) +	3.2774e−1 (1.51e−2)
	3	5.4550e−2 (7.96e−5) +	5.4534e−2 (6.27e−5) +	7.0869e−2 (6.84e−4) +	5.4527e−2 (4.82e−4) +	8.1252e−2 (1.12e−3) +	4.2993e−1 (3.32e−2)
DTLZ3	2	1.4814e + 0 (1.10e + 0) +	4.7188e + 0 (3.20e + 0) +	6.4932e−1 (8.40e−1) +	6.7843e−1 (8.81e−1) +	7.7134e−1 (5.98e−1) +	1.8142e + 2 (2.43e + 0)
	3	2.3425e + 0 (1.07e + 0) +	3.8616e + 0 (1.55e + 0) +	7.1857e−1 (5.58e−1) +	9.5987e−1 (5.90e−1) +	9.9734e−1 (7.84e−1) +	1.9941e + 2 (9.18e + 0)
DTLZ4	2	3.9734e−3 (2.49e−6) +	4.9135e−3 (3.60e−4) +	5.0529e−3 (1.11e−4) +	2.9934e−1 (4.04e−1) =	1.6155e−1 (3.25e−1) =	4.2833e−1 (1.16e−2)
	3	5.4529e−2 (1.59e−5) +	5.4578e−2 (1.45e−4) +	6.7868e−2 (3.07e−3) +	1.5184e−1 (2.17e−1) +	8.0769e−2 (4.18e−3) +	6.2438e−1 (2.17e−2)
DTLZ5	2	3.9725e−3 (2.08e−6) +	4.9054e−3 (7.08e−4) +	5.2364e−3 (3.42e−4) +	4.1861e−3 (3.20e−5) +	1.6419e−2 (2.30e−3) +	3.3372e−1 (3.35e−2)
	3	1.3187e−2 (7.86e−4) +	8.9849e−2 (2.91e−2) +	5.8433e−3 (3.67e−4) +	4.5769e−3 (1.52e−4) +	1.5419e−2 (1.63e−3) +	3.2330e−1 (3.22e−2)
DTLZ6	2	3.9671e−3 (3.66e−7) +	3.9695e−3 (2.31e−6) +	5.6765e−3 (2.94e−4) +	4.0804e−3 (1.95e−5) +	3.0707e−2 (1.72e−3) −	6.7043e−3 (4.10e−4)
	3	1.9953e−2 (1.48e−3) =	8.0223e−2 (1.36e−2) −	5.6950e−3 (2.18e−4) +	4.0860e−3 (2.48e−5) +	2.8297e−2 (3.20e−3) =	2.3471e−2 (8.33e−3)
DTLZ7	2	4.7668e−3 (9.20e−5) +	3.8324e−2 (1.08e−2) −	5.3869e−3 (1.81e−4) +	5.1999e−3 (1.11e−4) +	5.9739e−3 (1.29e−4) =	6.0539e−3 (3.22e−4)
	3	6.1886e−2 (1.22e−3) +	1.1173e−1 (3.72e−3) =	7.9862e−2 (3.67e−3) +	7.7680e−2 (2.16e−3) +	7.6276e−2 (3.03e−3) +	1.0524e−1 (8.02e−3)
CONSTR	2	2.7365e−2 (1.01e−3) +	1.4755e−1 (1.21e−2) +	3.6649e−2 (1.10e−2) +	2.4496e + 0 (2.53e−2) =	2.4406e + 0 (1.16e−2) =	2.4375e + 0 (1.90e−2)
TNK	2	4.5601e−3 (1.23e−4)	3.4399e−2 (3.12e−3)	5.7426e−3 (1.86e−4)	2.4346e + 0 (9.98e−3)	1.8222e−1 (4.93e−2) =	8.0958e + 1 (2.60e + 1)
SRN	2	1.1036e + 0 (3.20e−2)	8.6933e + 0 (1.02e + 0)	8.3189e−1 (2.74e−2)	1.0593e + 0 (1.13e−2)	3.1281e−2 (3.26e−3)	1.1286e + 0 (4.96e−2) =
OSY	2	7.4011e + 1 (4.09e + 1)	7.9798e + 1 (2.26e + 1)	8.8880e + 1 (3.25e + 1)	5.4045e−1 (9.42e−3) =	8.3425e + 0 (2.32e + 0)	2.7932e−1 (2.56e−2) =
BNH	2	5.0551e−1 (1.39e−2) =	1.0828e + 0 (2.61e−2) −	1.0555e + 0 (8.22e−3) −	4.1436e−1 (1.04e−2) +	9.7701e−1 (8.68e−2) −	5.1216e−1 (2.14e−2)
KITA	2	9.6365e−2 (9.04e−5) +	2.7341e−1 (4.95e−2) −	1.2728e−1 (6.18e−3) +	1.2914e−1 (1.24e−3) +	1.4123e−1 (3.15e−3) +	1.4993e−1 (5.05e−3)

#### Analysis using the spacing (SP) metric

Table 3 displays the final solution distributions when comparing MOEDO algorithm against MOMPA, NSGA-II, MOAOA, MOEA/D and MOGNDO using the spacing metric. MOEDO showcased commendable performance in ZDT1 for average metrics, while in ZDT4, it excelled in the average metric. For DTLZ 9/14, our method stood out in the standard metric. In the constraint benchmark, encompassing TNK, OSY, BNH and KITA, MOEDO consistently scored higher across all metrics. MOEA/D lagged behind in all benchmarks. The WRST test for the Spacing metric positioned MOEDO as the top performer, followed by MOGNDO and NSGA-II.

**Table 3:** Results of SP metric of different multi-objective algorithms on ZDT, DTLZ and constraint benchmark problems.

Problem	M	MOEDO	MOMPA	NSGA-II	MOAOA	MOEA/D	MOGNDO
ZDT1	2	7.1214e−3 (5.31e−4) +	1.6913e−2 (5.91e−3) −	1.0887e−2 (2.02e−3) =	7.6263e−3 (9.87e−4) +	7.5682e−3 (1.47e−3) +	1.0630e−2 (1.06e−3)
ZDT2	2	8.1044e−3 (5.95e−3) =	1.7679e−2 (9.02e−3) −	6.2838e−3 (4.02e−3) =	1.3871e−2 (4.17e−3) −	9.3412e−3 (5.31e−3) =	6.9593e−3 (3.58e−3)
ZDT3	2	6.3668e−3 (2.00e−3) +	3.5000e−2 (1.43e−2) −	1.1677e−2 (3.78e−3) =	5.1791e−3 (1.07e−3) +	2.9112e−2 (1.00e−2) −	1.0642e−2 (2.59e−3)
ZDT4	2	7.6587e−3 (5.43e−4) +	3.2109e−2 (2.55e−2) −	1.2094e−2 (3.15e−3) =	2.4643e−2 (7.84e−3) −	1.5679e−2 (1.05e−2) =	1.1124e−2 (1.21e−3)
ZDT5	2	1.0161e−2 (3.21e−2) +	1.8617e + 0 (3.36e−1) −	2.0357e−1 (3.62e−2) +	3.4202e−1 (7.62e−2) +	3.3569e−2 (7.08e−2) +	5.8873e−1 (2.67e−1)
ZDT6	2	5.5247e−3 (5.15e−4) −	1.9622e−2 (5.50e−3) −	4.9764e−3 (1.31e−3) −	8.9430e−3 (7.09e−4) −	6.4824e−3 (1.36e−3) −	3.7357e−3 (1.58e−3)
DTLZ1	2	1.5596e−3 (2.72e−4) +	2.4113e−2 (2.70e−2) +	7.2210e−4 (2.06e−4) +	3.3884e−3 (1.37e−4) +	1.5455e−1 (8.18e−2) +	5.5336e + 0 (2.68e + 0)
	3	8.5216e−3 (1.38e−3) +	4.9424e−2 (3.69e−2) +	1.3803e−2 (1.79e−2) +	1.9562e−2 (1.26e−3) +	9.3928e−2 (1.19e−1) +	4.6740e + 0 (1.45e + 0)
DTLZ2	2	3.4446e−3 (4.74e−4) +	6.4510e−3 (1.44e−3) +	6.2005e−3 (3.94e−5) +	6.7530e−3 (2.92e−4) +	2.6631e−2 (1.58e−3) +	1.7875e−1 (6.89e−2)
	3	2.3090e−2 (2.46e−3) +	5.6804e−2 (5.46e−4) +	5.7267e−2 (4.02e−4) +	5.5118e−2 (5.25e−3) +	6.6165e−2 (3.56e−3) +	1.6504e−1 (3.06e−2)
DTLZ3	2	7.0258e−2 (6.23e−2) +	5.5228e−1 (2.94e−1) +	2.6104e−1 (1.05e−1) +	6.7597e−2 (9.82e−2) +	8.9801e−1 (9.88e−1) +	3.7166e + 1 (1.69e + 1)
	3	2.6771e + 0 (5.21e + 0) +	6.2909e−1 (3.01e−1) +	3.4681e−1 (1.54e−1) +	9.4149e−2 (3.71e−2) +	7.0548e−1 (4.69e−1) +	3.5037e + 1 (8.89e + 0)
DTLZ4	2	1.6482e−2 (9.37e−3) +	5.7672e−3 (1.35e−4) +	6.1890e−3 (4.53e−5) +	7.1392e−3 (4.43e−4) +	NaN (NaN)	6.1747e−1 (1.42e−1)
	3	6.5210e−2 (5.36e−3) +	5.6773e−2 (1.67e−4) +	5.6725e−2 (3.09e−4) +	5.9118e−2 (2.52e−3) +	2.1135e−2 (1.02e−2) +	4.3779e−1 (2.08e−1)
DTLZ5	2	3.3436e−3 (5.03e−4) +	5.7156e−3 (5.67e−4) +	6.2012e−3 (4.57e−5) +	6.9006e−3 (7.07e−4) +	2.7132e−2 (2.82e−3) +	2.4357e−1 (7.95e−2)
	3	4.9022e−3 (1.02e−3) +	1.3195e−1 (6.07e−2) =	1.4769e−2 (3.25e−3) +	9.7820e−3 (7.85e−4) +	2.0880e−2 (5.77e−3) +	1.1697e−1 (1.01e−2)
DTLZ6	2	3.2839e−3 (1.53e−4) +	6.0118e−3 (7.08e−5) +	6.0678e−3 (2.51e−5) +	9.5433e−3 (8.22e−4) =	1.1594e−2 (1.42e−3) −	8.6874e−3 (5.23e−4)
	3	4.3746e−3 (3.93e−4) +	2.0032e−1 (8.13e−2) −	1.4425e−2 (3.55e−3) +	1.1627e−2 (8.52e−4) +	1.3992e−2 (1.25e−3) +	3.4821e−2 (1.55e−2)
DTLZ7	2	3.5729e−3 (5.84e−4) +	2.0634e−2 (2.99e−3) −	1.1228e−2 (8.61e−4) =	8.1454e−3 (4.27e−4) +	1.0782e−2 (4.42e−4) =	1.1565e−2 (1.16e−3)
	3	3.4012e−2 (3.74e−3) +	1.1205e−1 (4.70e−3) =	6.7504e−2 (4.98e−3) +	7.2390e−2 (2.72e−3) +	7.0513e−2 (4.85e−3) +	1.6617e−1 (4.89e−2)
CONSTR	2	4.5488e−2 (1.59e−3) −	1.6545e−1 (4.53e−2) −	5.2226e−2 (5.68e−3) −	2.2870e−2 (7.94e−3) =	2.1037e−2 (7.40e−3) =	1.6774e−2 (3.21e−3)
TNK	2	7.3809e−3 (4.18e−4)	3.6562e−2 (1.71e−2)	8.1623e−3 (5.99e−4)	1.1724e + 1 (1.07e + 1)	2.1316e−1 (4.26e−2) =	4.5412e−2 (4.09e−3) =
SRN	2	1.7219e + 0 (1.59e−1)	9.3200e + 0 (2.72e + 0)	9.9125e−1 (2.97e−1)	2.6179e + 0 (1.51e−1) =	3.0393e−2 (1.68e−3)	1.5422e + 0 (1.22e−1)
OSY	2	5.2872e−1 (3.88e−1)	1.4278e + 1 (1.32e + 1)	6.6767e−1 (5.04e−1)	2.9190e−1 (4.68e−2) =	8.1866e + 0 (4.31e + 0)	NaN (NaN)
BNH	2	3.4166e−1 (6.93e−2) +	2.5485e + 0 (6.08e−2) −	2.5404e + 0 (1.32e−2) −	7.8826e−1 (4.36e−2) =	9.9056e−1 (2.20e−1) =	2.9058e−1 (1.00e−1)
KITA	2	8.8811e−2 (7.64e−3) +	3.2781e−1 (6.20e−2) −	1.4685e−1 (6.04e−4) +	1.7733e−1 (2.09e−2) =	1.7994e−1 (1.70e−2) +	2.1774e−1 (3.18e−2)

#### Analysis using the spread (SD) metric

Table 4 presents the final solution distributions of MOEDO algorithm against MOMPA, NSGA-II, MOAOA, MOEA/D and MOGNDO using the Spread metric. MOEDO was dominant in ZDT2 across all metrics and in ZDT2, ZDT3, ZDT4 and ZDT5 for both the std and average metrics. In the DTLZ benchmark, our method was superior in 10/14 for both std and average metrics. For the constraint benchmark, MOEDO outshined in TNK and OSY for the standard metric. MOEA/D consistently underperformed. The WRST test, based on the results from Table 4, highlighted MOEDO as the leading method among the evaluated algorithms.

**Table 4:** Results of SD metric of different multi-objective algorithms on ZDT, DTLZ and constraint benchmark problems.

Problem	M	MOEDO	MOMPA	NSGA-II	MOAOA	MOEA/D	MOGNDO
ZDT1	2	4.4163e−1 (6.17e−2) +	6.8453e−1 (8.99e−2) −	5.9068e−1 (1.00e−1) =	4.1949e−1 (2.23e−2) +	5.8964e−1 (1.41e−1) =	5.6373e−1 (1.22e−1)
ZDT2	2	5.1648e−1 (1.41e−1) +	8.2618e−1 (1.18e−1) =	8.8220e−1 (6.38e−2) −	6.2752e−1 (1.35e−1) +	8.4278e−1 (2.14e−1) =	7.6411e−1 (1.32e−1)
ZDT3	2	6.1982e−1 (1.53e−1) =	7.0831e−1 (6.73e−2) =	6.8544e−1 (9.53e−2) =	7.9616e−1 (1.84e−2) −	8.9016e−1 (1.20e−1) −	7.3426e−1 (7.08e−2)
ZDT4	2	4.4500e−1 (5.26e−2) +	7.4246e−1 (1.76e−1) −	6.4107e−1 (1.58e−1) =	1.1363e + 0 (7.19e−2) −	1.1202e + 0 (2.25e−1) −	5.8509e−1 (1.05e−1)
ZDT5	2	7.7788e−1 (5.22e−2) +	1.0194e + 0 (6.14e−2) +	1.4541e + 0 (8.42e−2) +	1.3879e + 0 (7.66e−2) +	1.0090e + 0 (1.89e−2) +	1.5994e + 0 (5.47e−2)
ZDT6	2	3.6182e−1 (4.12e−2) −	5.5838e−1 (7.53e−2) −	3.1380e−1 (1.19e−1) −	4.3215e−1 (6.83e−2) −	2.9883e−1 (6.18e−2) −	1.9571e−1 (6.16e−2)
DTLZ1	2	1.3584e−1 (2.20e−2) +	6.5782e−1 (4.22e−1) =	6.8144e−2 (2.09e−2) +	3.7889e−1 (2.99e−2) +	1.1108e + 0 (5.62e−1) =	9.2205e−1 (1.44e−1)
	3	9.4036e−2 (1.84e−2) +	5.1939e−1 (4.05e−1) =	1.7879e−1 (2.42e−1) =	4.2387e−1 (2.89e−2) +	1.5185e + 0 (4.47e−1) −	5.7706e−1 (7.81e−2)
DTLZ2	2	1.4097e−1 (2.12e−2) +	1.9901e−1 (2.84e−2) +	1.9714e−1 (3.81e−3) +	3.6979e−1 (2.72e−2) +	7.0152e−1 (2.50e−2) =	8.1712e−1 (1.15e−1)
	3	8.5844e−2 (8.67e−3) +	1.7089e−1 (6.60e−4) +	1.7596e−1 (5.27e−3) +	5.0961e−1 (4.78e−2) =	4.5225e−1 (3.65e−2) +	6.2718e−1 (9.24e−2)
DTLZ3	2	7.9314e−1 (2.44e−1) =	8.5798e−1 (4.51e−2) =	1.0629e + 0 (1.46e−1) =	9.2082e−1 (2.29e−1) =	1.9088e + 0 (9.80e−1) −	9.2511e−1 (7.75e−2)
	3	1.1413e + 0 (5.29e−1) =	5.6418e−1 (9.72e−2) =	7.9222e−1 (4.53e−2) −	5.8120e−1 (9.30e−2) +	1.7308e + 0 (4.87e−1) −	6.8872e−1 (3.27e−2)
DTLZ4	2	1.5709e + 0 (7.99e−2)	1.8050e−1 (5.45e−3) +	1.9512e−1 (1.92e−3) +	4.0893e−1 (4.96e−2) +	6.8654e−1 (1.79e−1) +	NaN (NaN)
	3	1.3314e + 0 (1.90e−1)	1.7012e−1 (1.13e−3) +	1.7448e−1 (2.26e−3) +	4.9689e−1 (2.62e−2) +	4.8118e−1 (4.02e−2) +	1.9433e−1 (2.13e−1) +
DTLZ5	2	1.3710e−1 (2.87e−2) +	1.7534e−1 (1.56e−2) +	1.9703e−1 (1.63e−3) +	4.0634e−1 (4.82e−2) +	7.0571e−1 (3.73e−2) +	9.2448e−1 (1.13e−1)
	3	1.4378e−1 (2.25e−2) +	4.0977e−1 (1.32e−1) +	9.3751e−1 (1.14e−1) −	4.8666e−1 (4.28e−2) +	6.1465e−1 (3.99e−2) =	6.5765e−1 (5.69e−2)
DTLZ6	2	1.2150e−1 (1.45e−2) +	1.8776e−1 (2.64e−3) +	1.8805e−1 (1.08e−3) +	7.2139e−1 (7.40e−2) −	9.3383e−1 (5.00e−2) −	3.5990e−1 (3.16e−2)
	3	1.2833e−1 (1.03e−2) +	5.7465e−1 (1.59e−1) =	1.3798e + 0 (9.06e−2) −	7.2224e−1 (8.64e−2) −	9.2131e−1 (6.62e−2) −	4.2125e−1 (1.19e−1)
DTLZ7	2	1.2751e−1 (2.16e−2) +	4.3887e−1 (9.43e−2) =	5.6900e−1 (3.49e−2) =	4.2750e−1 (1.63e−2) +	4.3287e−1 (3.51e−2) +	5.1798e−1 (4.13e−2)
	3	1.2708e−1 (1.85e−2) +	3.6140e−1 (1.73e−2) +	6.1178e−1 (1.03e−1) =	4.9795e−1 (1.58e−2) +	5.1748e−1 (3.84e−2) =	5.8885e−1 (7.15e−2)
CONSTR	2	4.0032e−1 (1.17e−1) +	8.0320e−1 (4.41e−2) +	6.9753e−1 (1.31e−1) +	9.5806e−1 (7.00e−3) +	9.6345e−1 (1.03e−2) =	9.7633e−1 (3.88e−3)
TNK	2	5.4136e−1 (1.48e−1)	8.3119e−1 (3.05e−2)	1.0010e + 0 (8.65e−2)	8.4346e−1 (1.02e−1) =	1.0767e + 0 (3.07e−2)	9.7003e−1 (3.18e−3)
SRN	2	4.5643e−1 (5.49e−2)	4.4755e−1 (8.49e−2)	1.0556e−1 (2.35e−2)	7.3960e−1 (1.29e−1)	7.1203e−1 (2.51e−2) =	6.0084e−1 (4.71e−2) =
OSY	2	9.2304e−1 (6.48e−2)	9.6810e−1 (1.03e−1)	1.0009e + 0 (1.86e−2)	5.5471e−1 (5.68e−2) =	4.6138e−1 (5.00e−2) =	5.0966e−1 (4.10e−2)
BNH	2	5.1998e−1 (4.41e−2) =	6.9675e−1 (3.15e−2) −	6.7556e−1 (2.77e−3) −	1.4154e−1 (2.05e−2) +	5.6680e−1 (5.28e−2) −	4.7468e−1 (3.95e−2)
KITA	2	6.0594e−1 (6.55e−2) =	5.1226e−1 (8.49e−2) =	3.3402e−1 (7.78e−4) +	1.3280e−1 (1.07e−2) +	3.2786e−1 (4.70e−2) +	5.2172e−1 (6.67e−2)

#### Analysis using the HV metric

Table 5 delineates the final solution distributions when contrasting MOEDO algorithm against MOMPA, NSGA-II, MOAOA, MOEA/D and MOGNDO using the HV metric. Our method outshone in 15 out of the 26 problems. It was particularly dominant in ZDT3 and ZDT5 across metrics. In the DTLZ benchmark, MOEDO was unparalleled, especially in DTLZ4. Lastly, in the constraint test suites, MOEDO excelled in TNK, OSY and KITA across all metrics. In the ZDT benchmarks, MOAOA consistently underperformed. The WRST test outcomes for the HV metric positioned MOEDO in the second spot among the five evaluated multi-objective methods.

**Table 5:** Results of HV metric of different multi-objective algorithms on ZDT, DTLZ and constraint benchmark problems.

Problem	M	MOEDO	MOMPA	NSGA-II	MOAOA	MOEA/D	MOGNDO
ZDT1	2	8.6894e−1 (3.27e−3) +	7.0827e−1 (5.62e−2) −	8.3442e−1 (3.08e−2) =	8.6446e−1 (4.49e−3) +	8.3849e−1 (1.68e−2) =	8.4607e−1 (2.34e−2)
ZDT2	2	5.0055e−1 (6.08e−2) +	3.0232e−1 (8.18e−2) −	3.1603e−1 (6.09e−2) −	4.8074e−1 (4.52e−2) +	3.6941e−1 (1.53e−1) =	4.0078e−1 (8.79e−2)
ZDT3	2	9.2335e−1 (1.28e−1) =	7.8164e−1 (8.15e−2) −	9.8307e−1 (3.86e−2) =	8.5116e−1 (7.83e−2) =	9.4019e−1 (4.36e−2) =	9.2170e−1 (9.51e−2)
ZDT4	2	8.6455e−1 (2.96e−3) +	6.8366e−1 (1.34e−1) −	8.2530e−1 (4.02e−2) =	7.1103e−1 (1.05e−1) −	7.5134e−1 (8.52e−2) −	8.4269e−1 (2.46e−2)
ZDT5	2	3.0693e + 2 (6.60e + 0) =	3.0368e + 2 (3.61e + 0) =	3.0927e + 2 (5.59e + 0) =	3.0694e + 2 (5.22e + 0) =	2.7668e + 2 (2.69e + 0) −	3.0626e + 2 (3.06e + 0)
ZDT6	2	4.3088e−1 (3.53e−4) =	3.2899e−1 (3.00e−2) −	4.2327e−1 (6.42e−3) −	4.2961e−1 (2.12e−3) =	4.1510e−1 (5.64e−3) −	4.2963e−1 (2.49e−3)
DTLZ1	2	1.7473e−1 (1.38e−3) +	1.6326e−1 (9.97e−3) +	1.7498e−1 (9.36e−4) +	1.7399e−1 (1.13e−3) +	1.2496e−1 (7.28e−3) +	0.0000e + 0 (0.00e + 0)
	3	1.3865e−1 (7.84e−4) +	1.2212e−1 (1.93e−2) +	1.3806e−1 (1.82e−3) +	1.3637e−1 (5.14e−4) +	7.0145e−2 (1.47e−2) +	0.0000e + 0 (0.00e + 0)
DTLZ2	2	4.2015e−1 (1.04e−4) +	4.1779e−1 (1.04e−3) +	4.2006e−1 (2.29e−5) +	4.1924e−1 (5.40e−5) +	4.1885e−1 (1.61e−4) +	3.1484e−2 (2.30e−2)
	3	7.3819e−1 (1.81e−3) +	7.4349e−1 (5.65e−4) +	7.4399e−1 (1.66e−4) +	7.0703e−1 (5.66e−3) +	7.4192e−1 (1.19e−3) +	4.5622e−2 (1.76e−2)
DTLZ3	2	1.6041e−1 (1.66e−1) =	0.0000e + 0 (0.00e + 0) =	1.9760e−2 (3.06e−2) =	1.4198e−1 (1.37e−1) =	6.6897e−2 (6.89e−2) =	0.0000e + 0 (0.00e + 0)
	3	1.5867e−1 (3.03e−1) =	0.0000e + 0 (0.00e + 0) =	0.0000e + 0 (0.00e + 0) =	2.2703e−1 (3.12e−1) =	1.2688e−1 (1.23e−1) =	0.0000e + 0 (0.00e + 0)
DTLZ4	2	2.9614e−1 (1.70e−1) +	4.1845e−1 (4.58e−4) +	4.2006e−1 (1.38e−5) +	4.1935e−1 (1.48e−4) +	3.5713e−1 (1.38e−1) +	5.8848e−4 (1.32e−3)
	3	6.8187e−1 (1.23e−1) +	7.4351e−1 (3.34e−4) +	7.4383e−1 (1.53e−4) +	7.1324e−1 (5.58e−3) +	7.4188e−1 (1.85e−3) +	0.0000e + 0 (0.00e + 0)
DTLZ5	2	4.2004e−1 (1.93e−5) +	4.1872e−1 (8.75e−4) +	4.2021e−1 (1.04e−4) +	4.1929e−1 (4.53e−4) +	4.1884e−1 (4.05e−4) +	2.8442e−2 (2.23e−2)
	3	1.3267e−1 (1.37e−4) +	9.6369e−2 (1.11e−2) +	1.2892e−1 (5.18e−4) +	1.3243e−1 (4.35e−5) +	1.3215e−1 (2.06e−4) +	4.8987e−3 (6.73e−3)
DTLZ6	2	4.2056e−1 (4.41e−5) +	4.2013e−1 (2.75e−6) +	4.2013e−1 (7.29e−7) +	4.1906e−1 (2.86e−4) +	4.1435e−1 (5.56e−4) −	4.1844e−1 (3.91e−4)
	3	1.3315e−1 (2.15e−5) +	9.7542e−2 (9.62e−3) −	1.2677e−1 (1.18e−3) =	1.3271e−1 (1.23e−4) +	1.3021e−1 (7.23e−4) +	1.2738e−1 (2.11e−3)
DTLZ7	2	1.0098e + 0 (1.48e−4) +	9.2610e−1 (2.55e−2) −	1.0090e + 0 (3.84e−4) =	1.0091e + 0 (1.99e−4) =	1.0093e + 0 (1.28e−4) =	1.0089e + 0 (3.33e−4)
	3	1.6154e + 0 (5.00e−3) +	1.5112e + 0 (2.78e−2) =	1.5723e + 0 (7.69e−3) +	1.5694e + 0 (1.06e−2) +	1.6393e + 0 (3.87e−3) +	1.5294e + 0 (1.58e−2)
CONSTR	2	5.2265e + 0 (1.66e−3) +	4.9753e + 0 (2.08e−2) +	5.2160e + 0 (1.02e−2) +	3.6151e + 0 (1.38e−1) =	3.6562e + 0 (6.27e−2) =	3.6649e + 0 (1.04e−1)
TNK	2	5.2277e−1 (3.54e−4)	4.8054e−1 (3.87e−3)	5.2110e−1 (9.37e−4)	5.2209e + 0 (7.81e−4) =	4.9433e + 0 (9.11e−2) =	3.6805e + 0 (5.44e−2)
SRN	2	2.9871e + 4 (9.83e + 0)	2.6932e + 4 (4.68e + 2)	2.9990e + 4 (6.60e + 0)	3.0996e + 3 (3.87e + 2)	6.4222e + 3 (3.87e + 0) =	NaN (NaN)
OSY	2	6.3066e + 3 (3.98e + 3)	4.7546e + 3 (1.50e + 3)	4.8425e + 3 (3.16e + 3)	2.9885e + 4 (4.79e + 0)	NaN (NaN)	NaN (NaN)
BNH	2	6.4406e + 3 (6.99e−1) =	6.4265e + 3 (2.70e + 0) −	6.4413e + 3 (1.43e−1) =	6.4430e + 3 (2.35e + 0) =	6.4458e + 3 (9.71e−1) +	6.4406e + 3 (1.31e + 0)
KITA	2	4.9805e + 1 (2.43e−3) +	4.8246e + 1 (3.66e−1) −	4.9646e + 1 (2.78e−2) +	4.9577e + 1 (1.61e−2) +	4.9387e + 1 (8.00e−2) =	4.9423e + 1 (8.70e−2)

#### Analysis using the RT metric

Table 6 illustrates the final solution distributions of MOEDO algorithm against MOMPA, NSGA-II, MOAOA, MOEA/D and MOGNDO using the initial gap metric. MOEDO surpassed its counterparts in DTLZ3 and DTLZ5 across all metrics, namely Best, Avg and Std. In the constraint functions, our method was superior in CONSTR, TNK, SRN and KITA for the RT metric. MOEA/D consistently trailed its peers. The WRST test, based on the results from Table 6, positioned MOEDO at the best among the tested methods.

**Table 6:** Results of RT metric of different multi-objective algorithms on ZDT, DTLZ and constraint benchmark problems.

Problem	M	MOEDO	MOMPA	NSGA-II	MOAOA	MOEA/D	MOGNDO
ZDT1	2	1.0840e + 0 (1.40e−1)	1.4185e + 0 (1.81e−1) −	1.5848e + 0 (1.57e−1) −	3.6373e + 0 (4.15e−1) −	1.8603e + 1 (1.04e + 0) −	5.3035e + 0 (3.26e−1) −
ZDT2	2	1.3086e + 0 (1.17e−1)	1.3579e + 0 (1.82e−1) =	1.8077e + 0 (1.17e−1) −	3.4632e + 0 (1.57e−1) −	1.8507e + 1 (2.52e + 0) −	3.6988e + 0 (7.49e−1) −
ZDT3	2	1.4600e + 0 (3.81e−1) −	1.0732e + 0 (1.55e−1)	1.6045e + 0 (4.06e−1) −	3.5964e + 0 (3.52e−1) −	1.8544e + 1 (1.97e + 0) −	5.4055e + 0 (4.99e−1) −
ZDT4	2	1.2239e + 0 (7.44e−2) −	9.5238e−1 (5.83e−2)	1.4150e + 0 (1.23e−1) −	3.4015e + 0 (3.48e−1) −	1.8608e + 1 (1.99e + 0) −	5.2289e + 0 (1.95e + 0) −
ZDT5	2	9.5160e−1 (2.93e−1)	1.4136e + 0 (3.76e−1) −	1.7063e + 0 (2.22e−1) −	3.5809e + 0 (4.79e−1) −	1.6872e + 1 (1.72e + 0) −	5.8031e + 0 (6.80e−1) −
ZDT6	2	9.3844e−1 (7.17e−2)	1.2634e + 0 (1.22e−1) −	1.4590e + 0 (1.17e−1) −	3.5204e + 0 (4.66e−1) −	1.8614e + 1 (2.56e + 0) −	6.0524e + 0 (7.98e−1) −
DTLZ1	2	9.6031e−1 (2.84e−1) +	1.2525e + 0 (1.53e−1) +	1.4012e + 0 (1.83e−1) +	4.4339e + 0 (5.50e−1) +	4.1116e + 0 (4.39e−1) +	6.6382e + 0 (4.64e−1)
	3	9.0816e−1 (1.04e−1) +	1.2163e + 0 (4.15e−2) +	1.4483e + 0 (8.74e−2) +	5.4172e + 0 (4.22e−1) +	4.1937e + 0 (5.50e−1) +	6.7279e + 0 (1.84e−1)
DTLZ2	2	8.4328e−1 (5.64e−2) +	1.2513e + 0 (6.22e−2) +	1.5130e + 0 (2.09e−1) +	8.3898e + 0 (4.59e + 0) =	4.4058e + 0 (4.70e−1) +	6.6612e + 0 (3.84e−1)
	3	9.2135e−1 (3.19e−2) +	1.2750e + 0 (5.76e−2) +	1.5529e + 0 (6.47e−2) +	1.1309e + 1 (5.47e−1) −	4.6738e + 0 (1.81e−1) +	7.0132e + 0 (2.77e−1)
DTLZ3	2	1.2627e + 0 (4.72e−2) +	9.6584e−1 (2.79e−2) +	1.3033e + 0 (5.11e−2) +	3.3023e + 0 (1.48e−1) +	4.3152e + 0 (1.85e−1) +	6.5943e + 0 (1.26e−1)
	3	1.2558e + 0 (1.88e−2) +	9.3736e−1 (2.48e−2) +	1.3975e + 0 (2.26e−2) +	3.7878e + 0 (3.80e−1) +	4.3024e + 0 (1.18e−2) +	6.7236e + 0 (6.21e−2)
DTLZ4	2	8.3014e−1 (1.08e−2) +	1.2242e + 0 (4.21e−3) +	1.3975e + 0 (9.26e−3) +	4.8757e + 0 (1.45e + 0) +	4.3340e + 0 (2.17e−1) +	6.7796e + 0 (2.91e−1)
	3	1.2504e + 0 (7.73e−3) +	9.1272e−1 (5.36e−3) +	1.5403e + 0 (1.24e−2) +	1.0096e + 1 (2.16e + 0) =	4.5982e + 0 (2.41e−1) +	7.4664e + 0 (7.93e−1)
DTLZ5	2	1.2979e + 0 (1.52e−1) +	8.7998e−1 (8.02e−2) +	1.4981e + 0 (1.70e−1) +	6.3156e + 0 (3.02e−1) +	4.3694e + 0 (1.78e−1) +	6.8971e + 0 (2.28e−1)
	3	1.0506e + 0 (1.11e−2) +	8.9586e−1 (7.35e−3) +	2.1173e + 0 (6.75e−2) +	7.6273e + 0 (3.17e−1) =	4.6740e + 0 (1.90e−1) +	7.5965e + 0 (6.30e−1)
DTLZ6	2	9.1287e−1 (6.27e−2) +	1.2573e + 0 (5.08e−2) +	1.4153e + 0 (5.08e−2) +	1.0634e + 1 (3.59e−1) −	4.7006e + 0 (1.47e−2) +	7.5665e + 0 (8.67e−1)
	3	9.3459e−1 (4.25e−2) +	1.2374e + 0 (6.09e−2) +	2.3709e + 0 (2.52e−1) +	1.0866e + 1 (7.69e−1) −	4.7894e + 0 (5.31e−2) +	7.3765e + 0 (2.24e−1)
DTLZ7	2	9.2944e−1 (4.34e−2) +	1.3029e + 0 (6.27e−2) +	1.4993e + 0 (7.73e−2) +	5.6874e + 0 (3.65e−1) +	4.5383e + 0 (2.13e−1) +	6.7145e + 0 (1.34e−1)
	3	9.6492e−1 (1.08e−2) +	1.1815e + 0 (4.01e−2) +	1.7620e + 0 (1.02e−1) +	9.4474e + 0 (3.09e−1) −	4.8045e + 0 (1.24e−2) +	7.0641e + 0 (1.52e−1)
CONSTR	2	8.7374e−1 (1.11e−1) +	1.2232e + 0 (7.82e−2) +	1.5144e + 0 (9.54e−2) +	1.3907e + 1 (6.31e−1) −	3.5439e + 0 (2.53e−1) +	6.3507e + 0 (1.61e−1)
TNK	2	8.6013e−1 (5.32e−2) +	1.2835e + 0 (1.17e−1) +	1.4880e + 0 (8.44e−2) +	1.1838e + 1 (4.76e−1) −	3.1048e + 0 (1.31e−1) +	6.3953e + 0 (2.79e−1)
SRN	2	8.4685e−1 (3.34e−2) +	1.1482e + 0 (7.09e−2) +	1.4259e + 0 (7.34e−2) +	1.3554e + 1 (1.67e−1) −	3.5035e + 0 (6.69e−2) +	6.1560e + 0 (1.24e−1)
OSY	2	1.2234e + 0 (6.40e−2) +	9.1941e−1 (4.61e−2) +	1.4842e + 0 (8.07e−2) +	7.1965e + 0 (2.63e−1) −	3.4530e + 0 (1.37e−1) +	6.0350e + 0 (1.88e−1)
BNH	2	1.2135e + 0 (4.57e−2) +	7.8729e−1 (1.44e−2) +	1.3896e + 0 (1.18e−2) +	1.1663e + 1 (1.46e−1) −	3.4872e + 0 (2.42e−2) +	6.0393e + 0 (2.19e−1)
KITA	2	8.4522e−1 (5.03e−2) +	1.1953e + 0 (7.01e−2) +	1.4823e + 0 (1.28e−1) +	1.3566e + 1 (4.96e−1) −	3.4910e + 0 (1.98e−2) +	6.1794e + 0 (1.93e−1)

**Figure 6:**
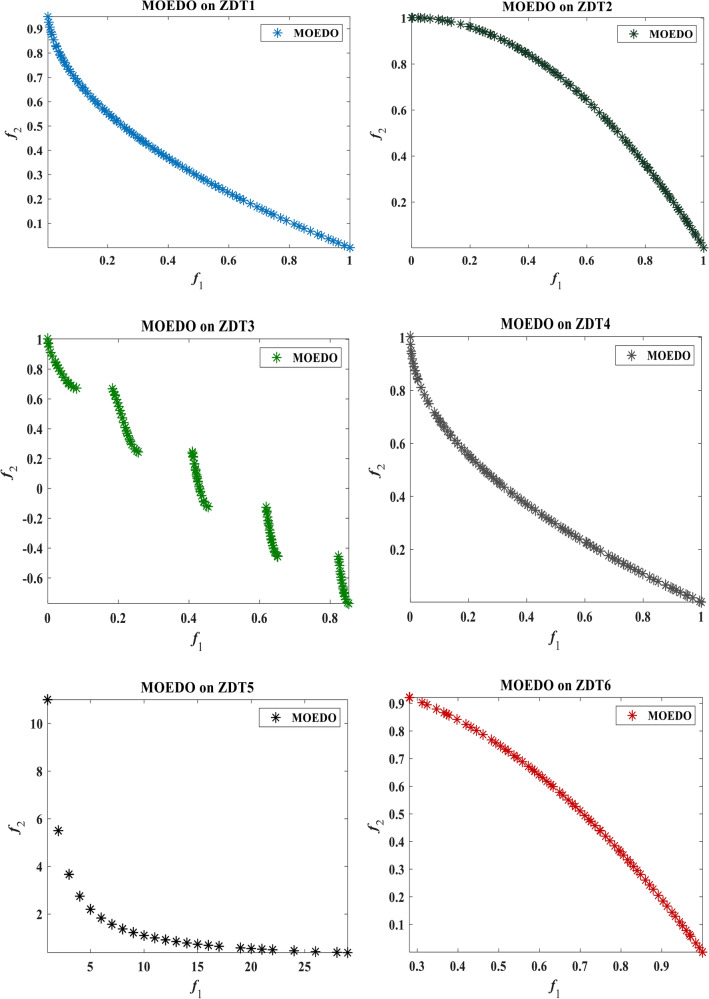
Best Pareto optimal front obtained by the MOEDO algorithm on ZDT1, ZDT2, ZDT3, ZDT4, ZDT5 and ZDT6 problems.

**Figure 7:**
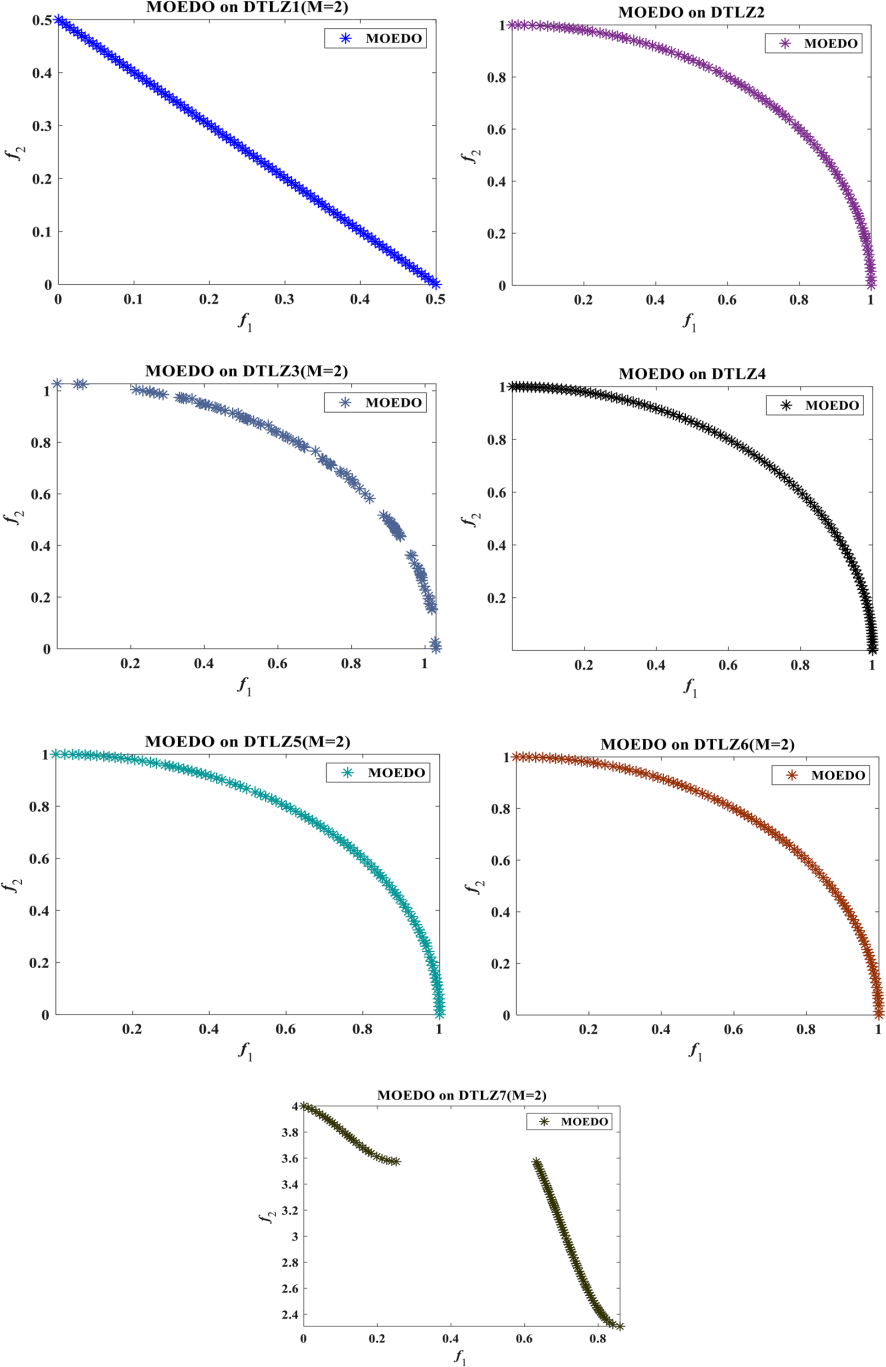
Best Pareto optimal front obtained by the MOEDO algorithm on DTLZ1-DTLZ7 problems with 2-objectives.

**Figure 8:**
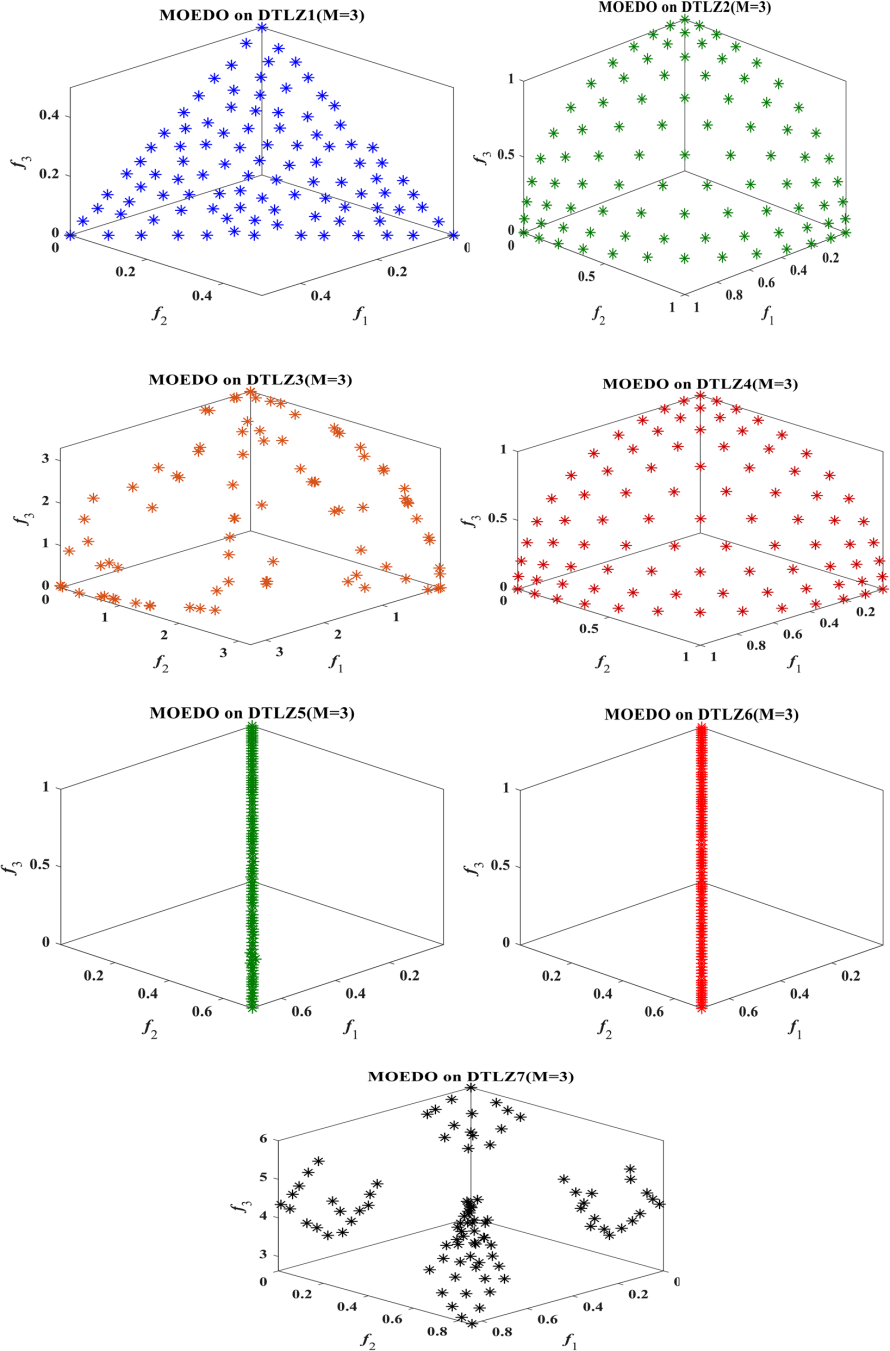
Best Pareto optimal front obtained by the MOEDO algorithm on DTLZ1-DTLZ7 problems with 3-objectives.

**Figure 9:**
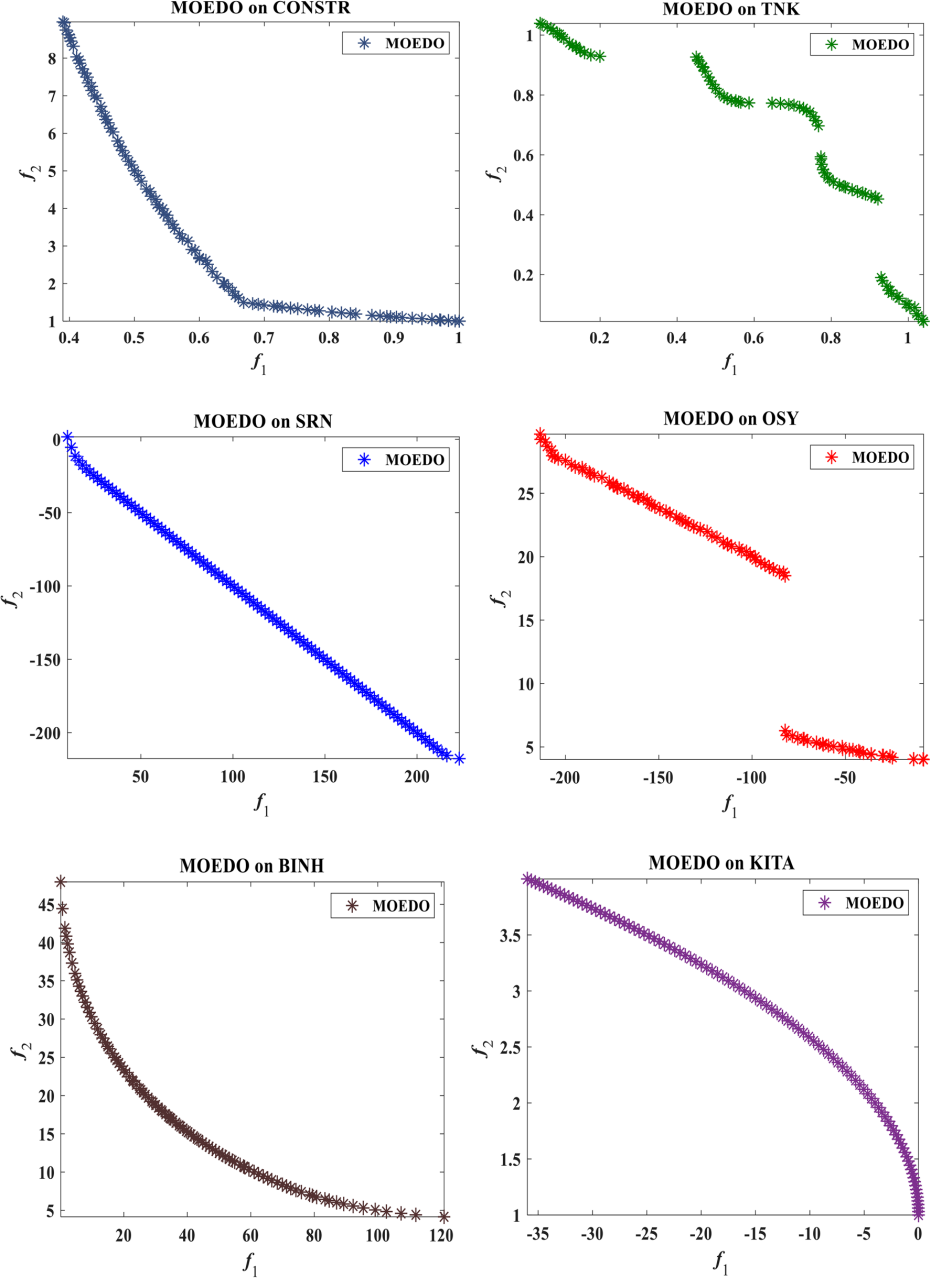
Best Pareto optimal front obtained by the MOEDO algorithm on constrained CONSTR, TANK, SRN, OSY, BIN and KITA.

The visual representation of the final solutions from our proposed MOEDO method. Figures 6, 7, 8 and 9 reveals MOEDO superior PF. This section provides a thorough assessment of MOEDO performance against leading multi-objective methods using metrics like GD, IGD, HV, Spacing, Spread and RT across standard test suites such as ZDT, Constraint and DTLZ. MOEDO commendable performance is attributed to its rapid convergence and a harmonious balance between exploration and exploitation. This is achieved by integrating the strengths of the IFM technique, the adept search capabilities of the EDO algorithm and the enhanced convergence facilitated by a IFM mechanism. Conversely, many of the compared multi-objective methods struggled with maintaining a balanced exploration–exploitation ratio and lacked efficient convergence.

### Application to real-world engineering design problems

Table 7 presents the performance of MOEDO concerning the spacing metric, compared against MOMPA, NSGA-II, MOAOA, MOEA/D and MOGNDO. A lower spacing value denotes superior coverage performance. The data reveals MOEDO performance RWMOP1, RWMOP2, RWMOP3 and RWMOP4 was suboptimal. The WRST test, as illustrated in Table 7, positions MOEDO as the top performer in terms of spacing values among all others.

**Table 7:** Results of SP metric of different multi-objective algorithms on engineering design problems.

Problem	M	MOEDO	MOMPA	NSGA-II	MOAOA	MOEA/D	MOGNDO
RWMOP1	2	1.8684e−2 (2.56e−3)	1.5023e−1 (5.14e−2)	2.2250e−2 (3.43e−3)	1.8791e−2 (2.20e−3)	1.9296e−2 (1.16e−3)	NaN (NaN)
RWMOP2	2	6.8240e + 2 (1.48e + 2) =	NaN (NaN)	1.0591e + 3 (2.04e + 2) =	1.1011e + 3 (2.48e + 2) =	7.9174e + 2 (2.86e + 2) =	7.0175e + 3 (1.14e + 4)
RWMOP3	2	5.5089e + 2 (4.58e + 1) +	2.5135e + 3 (9.34e + 2) =	3.1492e + 3 (3.34e + 2) =	1.6276e + 3 (9.43e + 2) =	5.6910e + 2 (1.59e + 1) +	4.4510e + 3 (6.57e + 3)
RWMOP4	2	2.1262e−1 (5.55e−3) +	NaN (NaN)	3.8102e−1 (2.55e−2) +	4.5326e−1 (1.16e−1) +	1.9114e−1 (1.52e−2) +	1.6764e + 0 (7.48e−1)
RWMOP5	2	6.9979e−2 (2.13e−3) +	4.3162e−1 (6.93e−2) =	9.5075e−2 (2.19e−2) +	1.4909e−1 (3.01e−2) +	9.3591e−2 (3.20e−2) +	1.6943e + 0 (1.70e + 0)

Table 8 showcases the findings of MOEDO for the HV metric, juxtaposed against other algorithms. A higher HV value is indicative of better performance. The data suggests that MOEDO excels across RWMOP1, RWMOP2, RWMOP5 and RWMOP6. However, the WRST test results in Table 8 indicate room for improvement in the HV metric for MOEDO.

**Table 8:** Results of HV metric of different multi-objective algorithms on engineering design problems.

Problem	M	MOEDO	MOMPA	NSGA-II	MOAOA	MOEA/D	MOGNDO
RWMOP1	2	7.8729e−1 (1.44e−2) +	0.0000e + 0 (0.00e + 0)	0.0000e + 0 (0.00e + 0)	0.0000e + 0 (0.00e + 0)	0.0000e + 0 (0.00e + 0)	NaN (NaN)
RWMOP2	2	3.4457e + 6 (6.45e + 3) +	2.2454e + 6 (1.45e + 6) =	3.4342e + 6 (1.42e + 4) +	3.4334e + 6 (1.22e + 4) +	3.4370e + 6 (9.58e + 3) +	1.7023e + 6 (6.62e + 5)
RWMOP3	2	4.6805e + 3 (1.54e + 0) +	4.5295e + 3 (2.76e + 1) =	4.6374e + 3 (9.37e + 0) +	4.6285e + 3 (1.39e + 1) +	4.6825e + 3 (1.23e + 0) +	3.6036e + 3 (1.69e + 3)
RWMOP4	2	8.9567e−1 (2.33e−3) +	4.5704e−1 (3.02e−1) =	8.8959e−1 (4.89e−3) +	8.9057e−1 (7.52e−3) +	8.9203e−1 (5.88e−3) +	7.2647e−1 (8.24e−2)
RWMOP5	2	4.2559e + 1 (2.31e−1) +	4.1280e + 1 (7.10e−2) +	4.1229e + 1 (1.71e−1) =	4.2041e + 1 (3.66e−1) +	4.2287e + 1 (4.05e−1) +	4.0906e + 1 (2.24e−1)

Table 9 showcases the performance of MOEDO concerning the run time metric, juxtaposed against other MOEDO others MOMPA, NSGA-II, MOAOA, MOEA/D and MOGNDO. Ideally, a lower RT metric value is preferred. Specifically, it showcased superior results for RWMOP2, RWMOP3, RWMOP4 and RWMOP5 in both Average and Best metrics. The overall ranking of each algorithm, based on the Best and Avg results, was determined using the WRST test. As illustrated in the concluding row of Table 5, MOEDO emerged as the top performer for the RT metric among the others.

**Table 9:** Results of RT metric of different multi-objective algorithms on engineering design problems.

Problem	M	MOEDO	MOMPA	NSGA-II	MOAOA	MOEA/D	MOGNDO
RWMOP1	2	1.8374e + 0 (1.28e−1) +	1.8087e + 0 (6.67e−2) +	2.7699e + 0 (1.66e−1) =	7.8034e + 0 (8.10e−1) −	1.8801e + 1 (1.11e + 0) −	2.5022e + 0 (1.69e−1)
RWMOP2	2	8.0916e−1 (4.71e−2) +	9.5380e−1 (4.64e−2) +	1.6790e + 0 (1.00e−1) +	2.1577e + 0 (5.26e−1) +	1.6126e + 1 (7.86e−1) −	3.5721e + 0 (2.56e−1)
RWMOP3	2	6.3854e−1 (2.10e−2) +	8.6947e−1 (2.00e−2) +	1.4797e + 0 (1.13e−2) +	7.8338e + 0 (3.47e−1) +	1.5504e + 1 (4.48e−1) =	1.5660e + 1 (1.65e + 0)
RWMOP4	2	9.5050e−1 (8.04e−2) +	9.8713e−1 (5.29e−2) +	1.7186e + 0 (1.04e−1) +	8.3498e + 0 (4.72e−1) =	1.6635e + 1 (8.28e−1) −	7.1340e + 0 (1.02e + 0)
RWMOP5	2	7.0057e−1 (3.21e−2) +	1.0097e + 0 (4.30e−2) +	1.7758e + 0 (7.51e−2) +	1.1412e + 1 (1.35e + 0) +	1.5428e + 1 (2.30e−1) =	1.5480e + 1 (4.85e−1)

**Figure 10:**
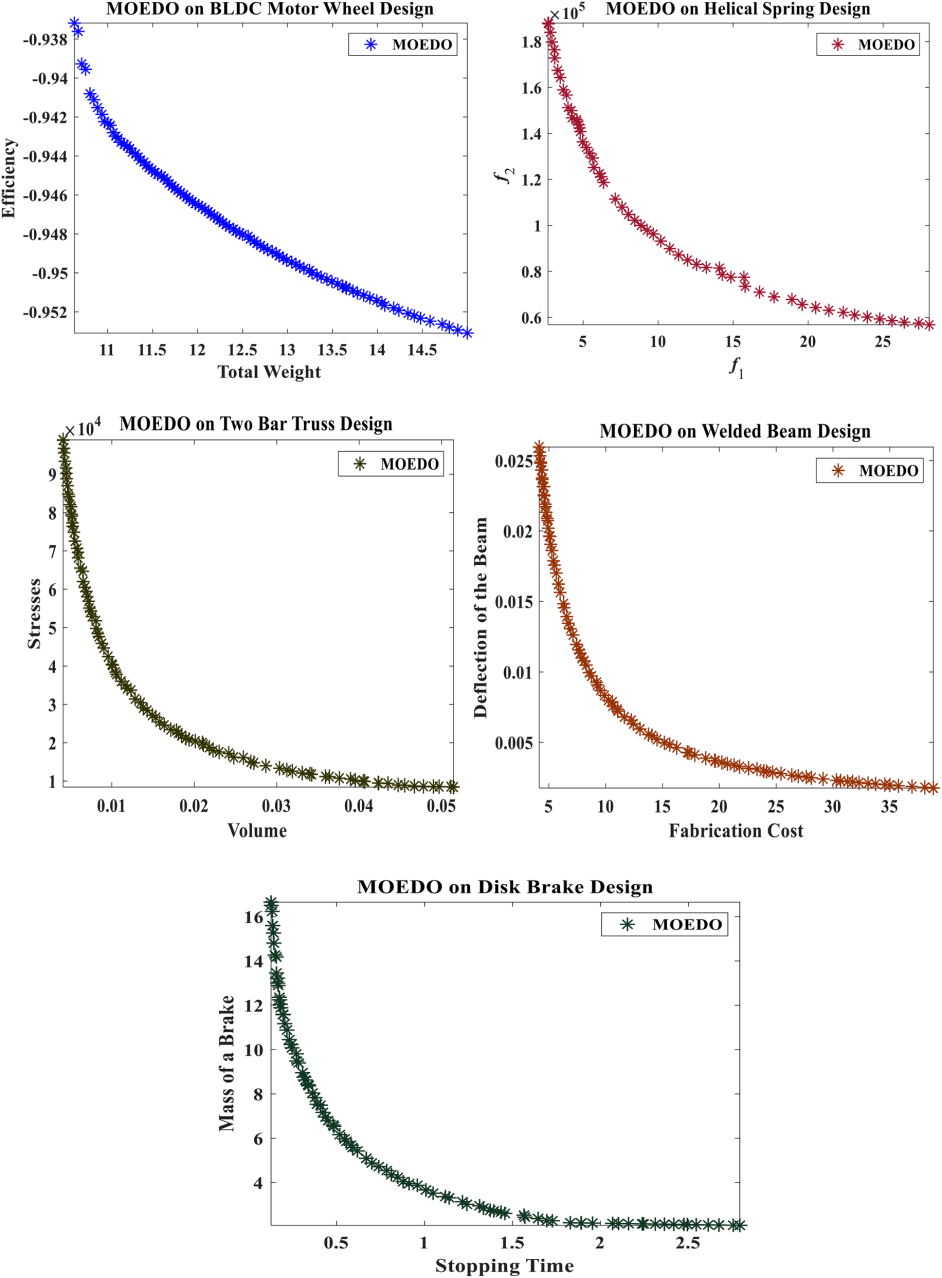
Best Pareto optimal front obtained by the MOEDO algorithm on real-world engineering problems (RWMOP1-RWMOP5).

Figure 10 emphasizes MOEDO superior performance for the RWMOP function, as its results closely align with the PF.

## Conclusion

In this research, we introduce a multi-objective variant of the exponential distribution optimizer (EDO) method, a recently proposed metaheuristic algorithm rooted in specific principles of mathematics exponential distribution theory. We present a multi-objective EDO termed MOEDO, which integrates concepts of multi-objectivity, NDS, CD and IFM theory into the conventional EDO framework. The IFM approach, drawing from diverse strategies, ensures a balanced exploration–exploitation dynamic, promoting enhanced convergence and the ability to bypass local minima. experimental outcomes indicate that our MOEDO algorithm surpasses MOMPA, NSGA-II, MOAOA, MOEA/D and MOGNDO, in 72.58% of test scenarios using the ZDT, DTLZ. Constraint (CONSTR, TNK, SRN, BNH, OSY and KITA) and real-world engineering design Brushless DC wheel motor (RWMOP1), Helical spring (RWMOP2), Two-bar truss (RWMOP3), Welded beam (RWMOP4), Disk brake (RWMOP5) problems, especially in metrics like GD, IGD, HV, spacing (SP), Spread (SD) and RT. In the WRST test outcomes, MOEDO leads in all metrics. Looking ahead, we envision developing a binary version of MOEDO to tackle diverse and intricate real-world challenges. Additionally, the potential enhancements of the proposed method in its many-objective format for various optimization challenges remain an exciting avenue for future exploration.The MOEDO source code is available at: https://github.com/kanak02/MOEDO.

### Supplementary Information

Supplementary Information.

## Data Availability

The data presented in this study are available through email upon request to the corresponding author.

## References

[1] Shi M, Lv L, Xu L (2023). A multi-fidelity surrogate model based on extreme support vector regression: Fusing different fidelity data for engineering design. Eng. Comput..

[2] Zhou S, Zhang W, Jiang J, Zhong W, Gu J, Zhu W (2022). On the convergence of stochastic multi-objective gradient manipulation and beyond. Adv. Neural. Inf. Process. Syst..

[3] Cao B, Zhao J, Gu Y, Ling Y, Ma X (2020). Applying graph-based differential grouping for multiobjective large-scale optimization. Swarm Evol. Comput..

[4] Zhu B, Sun Y, Zhao J, Han J, Zhang P, Fan T (2023). A critical scenario search method for intelligent vehicle testing based on the social cognitive optimization algorithm. IEEE Trans. Intell. Transp. Syst..

[5] Cao B, Wang X, Zhang W, Song H, Lv Z (2020). A many-objective optimization model of industrial Internet of things based on private blockchain. IEEE Netw..

[6] Zhang C, Zhou L, Li Y (2023). Pareto optimal reconfiguration planning and distributed parallel motion control of mobile modular robots. IEEE Trans. Ind. Electron..

[7] Li S, Chen H, Chen Y, Xiong Y, Song Z (2023). Hybrid method with parallel-factor theory, a support vector machine, and particle filter optimization for intelligent machinery failure identification. Machines.

[8] Zhang L, Sun C, Cai G, Koh LH (2023). Charging and discharging optimization strategy for electric vehicles considering elasticity demand response. eTransportation.

[9] Cao B, Zhao J, Yang P, Gu Y, Muhammad K, Rodrigues JJPC, de Albuquerque VHC (2020). Multiobjective 3-D topology optimization of next-generation wireless data center network. IEEE Trans. Ind. Inform..

[10] Duan Y, Zhao Y, Hu J (2023). An initialization-free distributed algorithm for dynamic economic dispatch problems in microgrid: Modeling, optimization and analysis. Sustain. Energy Grids Netw.orks.

[11] Almufti SM (2019). Historical survey on metaheuristics algorithms. Int. J. Sci. World.

[12] Alorf A (2023). A survey of recently developed metaheuristics and their comparative analysis. Eng. Appl. Artif. Intell..

[13] Dokeroglu T, Sevinc E, Kucukyilmaz T, Cosar A (2019). A survey on new generation metaheuristic algorithms. Comput. Ind. Eng..

[14] Zhou A, Qu B-Y, Li H, Zhao S-Z, Suganthan PN, Zhang Q (2011). Multiobjective evolutionary algorithms: A survey of the state of the art. Swarm Evol. Comput..

[15] Hu, X., & Eberhart, R. Multiobjective optimization using dynamic neighborhood particle swarm optimization. In *Proceedings of the 2002 Congress on Evolutionary Computation*, *2* (pp. 1677–1681). CEC'02. IEEE Publications (Cat. *No. 02TH8600*) (2002).

[16] Gunantara N (2018). A review of multi-objective optimization: Methods and its applications. Cogent Eng..

[17] Sharma S, Kumar V (2022). A comprehensive review on multi-objective optimization techniques: Past, present and future. Arch. Comput. Methods Eng..

[18] Pereira JLJ, Oliver GA, Francisco MB, Cunha SS, Gomes GF (2021). A review of multi-objective optimization: Methods and algorithms in mechanical engineering problems. Arch. Comput. Methods Eng..

[19] Huy THB, Nallagownden P, Truong KH, Kannan R, Vo DN, Ho N (2022). Multi-objective search group algorithm for engineering design problems. Appl. Soft Comput..

[20] Li Y-J, Li H-N (2018). Interactive evolutionary multi-objective optimization and decision-making on life-cycle seismic design of bridge. Adv. Struct. Eng..

[21] Zhang, J., & Xing, L. A survey of multiobjective evolutionary algorithms. In *IEEE International Conference on Computational Science and Engineering (CSE) and IEEE International Conference on Embedded and Ubiquitous Computing*, Vol. 1, 93–100 (IEEE Publications, 2017). 10.1109/CSE-EUC.2017.27.

[22] Guliashki V, Toshev H, Korsemov C (2009). Survey of evolutionary algorithms used in multiobjective optimization. Probl. Eng. Cybern. Robot..

[23] Wang J, Su Y, Lin Q, Ma L, Gong D, Li J, Ming Z (2020). A survey of decomposition approaches in multiobjective evolutionary algorithms. Neurocomputing.

[24] Mashwani WK (2011). Hybrid multiobjective evolutionary algorithms: A survey of the state-of-the-art. Int. J. Comput. Sci. Issues.

[25] Xu, Q., Xu, Z., & Ma, T. (2019). A short survey and challenges for multiobjective evolutionary algorithms based on decomposition. In *International Conference on Computer, Information and Telecommunication Systems, CITS, IEEE*, 1–5 (*2019*). 10.1109/CITS.2019.8862046.

[26] Igel C (2014). No free lunch theorems: Limitations and perspectives of metaheuristics. Theory and Principled Methods for the Design of Metaheuristics.

[27] Chopard B, Tomassini M (2018). Performance and limitations of metaheuristics. An Introduction to Metaheuristics for Optimization.

[28] Dorigo M, Stützle T (2003). The ant colony optimization metaheuristic: Algorithms, applications and advances. Handbook of Metaheuristics.

[29] Marca, Y., Aguirre, H., Zapotecas, S., Liefooghe, A., Derbel, B., Verel, S., & Tanaka, K. Pareto dominance-based MOEAs on problems with difficult pareto set topologies. In *Proceedings of the Genetic and Evolutionary Computation Conference Companion*, 189–190 (2018). 10.1145/3205651.3205746.

[30] Zhang Q, Li H, MOEA/D (2007). MOEA/D: A multiobjective evolutionary algorithm based on decomposition. IEEE Trans. Evol. Comput..

[31] Khodadadi N, Talatahari S, DadrasEslamlou A (2022). MOTEO: A novel multi-objective thermal exchange optimization algorithm for engineering problems. Soft Comput..

[32] Houssein EH, Çelik E, Mahdy MA, Ghoniem RM (2022). Self-adaptive equilibrium optimizer for solving global, combinatorial, engineering and multi-objective problems. Expert Syst. Appl..

[33] Lin A, Yu P, Cheng S, Xing L (2022). One-to-one ensemble mechanism for decomposition-based multi-objective optimization. Swarm Evolut. Comput..

[34] Zheng J, Zhang Z, Zou J, Yang S, Ou J, Hu Y (2022). A dynamic multiobjective particle swarm optimization algorithm based on adversarial decomposition and neighborhood evolution. Swarm Evolut. Comput..

[35] Ben-Said A, Moukrim A, Guibadj RN, Verny J (2022). Using decompositionbased multi-objective algorithm to solve selective pickup and delivery problems with time windows. Comput. Oper. Res..

[36] Zouache D, Abdelaziz FB (2022). Guided manta ray foraging optimization using epsilon dominance for multi-objective optimization in engineering design. Expert Syst. Appl..

[37] Yin S, Luo Q, Zhou Y (2022). IBMSMA: An indicator-based multi-swarm slime mould algorithm for multi-objective truss optimization problems. J. Bionic Eng..

[38] Zitzler E, Künzli S (2004). Indicator-based selection in multiobjective search. PPSN.

[39] Abdi Y, Feizi-Derakhshi M-R (2020). Hybrid multi-objective evolutionary algorithm based on search manager framework for big data optimization problems. Appl. Soft Comput..

[40] Dutta S, Mallipeddi R, Das KN (2022). Hybrid selection based multi/manyobjective evolutionary algorithm. Sci. Rep..

[41] Kalita K, Pal S, Haldar S, Chakraborty S (2022). A hybrid TOPSIS-PR-GWO approach for multi-objective process parameter optimization. Process Integrat. Optim. Sustain..

[42] Chennuru VK, Timmappareddy SR (2022). Simulated annealing based undersampling (SAUS): A hybrid multi-objective optimization method to tackle class imbalance. Appl. Intell..

[43] Mirjalili S, Jangir P, Saremi S (2017). Multi-objective ant lion optimizer: A multi-objective optimization algorithm for solving engineering problems. Appl. Intell..

[44] Premkumar M, Jangir P, Sowmya R, Alhelou HH, Mirjalili S, Kumar BS (2021). Multi-objective equilibrium optimizer: Framework and development for solving multi-objective optimization problems. J. Comput. Design Eng..

[45] Premkumar M, Jangir P, Sowmya R, Alhelou HH, Heidari AA, Chen H (2020). MOSMA: Multi-objective slime mould algorithm based on elitist non-dominated sorting. IEEE Access.

[46] Premkumar M, Jangir P, Santhosh Kumar B, Sowmya R, HaesAlhelou H, Abualigah L, Yildiz AR, Mirjalili S (2021). A new arithmetic optimization algorithm for solving real-world multiobjective CEC-2021 constrained optimization problems: Diversity analysis and validations. IEEE Access.

[47] Buch H, Trivedi IN (2020). A new non-dominated sorting ions motion algorithm: Development and applications. Decis. Sci. Lett..

[48] Jangir P, Buch H, Mirjalili S, Manoharan P (2021). MOMPA: Multi-objective marine predator algorithm for solving multi-objective optimization problems. Evolut. Intell..

[49] Mirjalili S, Jangir P, Mirjalili SZ, Saremi S, Trivedi IN (2017). Optimization of problems with multiple objectives using the multi-verse optimization algorithm. Knowl. Based Syst..

[50] Jangir P, Jangir N (2018). A new non-dominated sorting grey wolf optimizer (NS-GWO) algorithm: Development and application to solve engineering designs and economic constrained emission dispatch problem with integration of wind power. Eng. Appl. Artif. Intell..

[51] Premkumar M, Jangir P, Sowmya R (2021). MOGBO: A new multiobjective gradient-based optimizer for real-world structural optimization problems. Knowl. Based Syst..

[52] Kumar S, Jangir P, Tejani GG, Premkumar M, Alhelou HH (2021). MOPGO: A new physics-based multi-objective plasma generation optimizer for solving structural optimization problems. IEEE Access.

[53] Jangir P, Heidari AA, Chen H (2021). Elitist non-dominated sorting Harris hawks optimization: Framework and developments for multi-objective problems. Expert Syst. Appl..

[54] Kumar S, Jangir P, Tejani GG, Premkumar M (2022). MOTEO: A novel physics-based multiobjective thermal exchange optimization algorithm to design truss structures. Knowl. Based Syst..

[55] Kumar S, Jangir P, Tejani GG, Premkumar M (2022). A decomposition based multi-objective heat transfer search algorithm for structure optimization. Knowl. Based Syst..

[56] Ganesh N, Shankar R, Kalita K, Jangir P, Oliva D, Pérez-Cisneros M (2023). A novel decomposition-based multi-objective symbiotic organism search optimization algorithm. Mathematics.

[57] Pandya SB, Visumathi J, Mahdal M, Mahanta TK, Jangir P (2022). A novel MOGNDO algorithm for security-constrained optimal power flow problems. Electronics.

[58] Jangir P (2018). Non-dominated sorting moth flame optimizer: A novel multi-objective optimization algorithm for solving engineering design problems. Eng. Technol. Open Access J..

[59] Jangir P, Jangir N (2017). Non-dominated sorting whale optimization algorithm. Glob. J. Res. Eng..

[60] Jangir P (2020). ‘MONSDA:-A novel multi-objective non-dominated sorting dragonfly algorithm’. glob. J. Res. Eng. F Electr. Electron. Eng..

[61] Jiao K, Chen J, Xin B, Li L (2023). A reference vector based multiobjective evolutionary algorithm with Q-learning for operator adaptation. Swarm Evolut. Comput..

[62] Li, C., Deng, L., Gong, W., & Qiao, L. A many-objective evolutionary algorithm based on hybrid dynamic decomposition IEEE Congress on Evolutionary Computation (CEC), 2023, 1–8 (IEEE Publications, 2023). 10.1109/CEC53210.2023.10254128.

[63] Pang LM, Ishibuchi H, Shang K (2023). Use of two penalty values in multiobjective evolutionary algorithm based on decomposition. IEEE Trans. Cybern..

[64] Abdel-Basset M, El-Shahat D, Jameel M, Abouhawwash M (2023). Exponential distribution optimizer (EDO): A novel math-inspired algorithm for global optimization and engineering problems. Artif. Intell. Rev..

[65] Zitzler E, Deb K, Thiele L (2000). Comparison of multiobjective evolutionary algorithms: Empirical results. Evol. Comput..

[66] Deb K, Thiele L, Laumanns M, Zitzler E (2005). Scalable test problems for evolutionary multiobjective optimization. Evolutionary Multiobjective Optimization.

[67] Binh, T. T., & Korn, U. MOBES: A multiobjective evolution strategy for constrained optimization problems. In *The Third International Conference on Genetic Algorithms (Mendel 97)*, 27 (1997).

[68] Osyczka A, Kundu S (1995). A new method to solve generalized multicriteria optimization problems using the simple genetic algorithm. Struct. Optim..

[69] Branke J, Kaußler T, Schmeck H (2001). Guidance in evolutionary multi-objective optimization. Adv. Eng. Softw..

[70] De la Hoz E, de la Hoz E, Ortiz A, Ortega J, Martínez-Álvarez A (2014). Feature selection by multi-objective optimisation: Application to network anomaly detection by hierarchical self-organising maps. Knowl. Based Syst..

[71] Martínez-Álvarez A, Cuenca-Asensi S, Ortiz A, Calvo-Zaragoza J, VivasTejuelo LAV (2015). Tuning compilations by multi-objective optimization: Application to apache web server. Appl. Soft Comput..

[72] Wang GG, Tan Y (2019). Improving metaheuristic algorithms with information feedback models. IEEE Trans. Cybern..

