# Supplementary material for: Multi-objective exponential distribution optimizer (MOEDO): a novel math-inspired multi-objective algorithm for global optimization and real-world engineering design problems
Source: Sci Rep. 2024 Jan 20;14:1816. doi: 10.1038/s41598-024-52083-7 (PMC10799915; doi:10.1038/s41598-024-52083-7)
Supplement: Supplementary file 1 — Supplementary Information. [file 41598_2024_52083_MOESM1_ESM.docx]

**Appendix A: Unconstrained multi-objective ZDT [62] test problems utilized in this work.**

A.1 ZDT1 with Convex POF:

$$F=\left( f_{1}\left( x \right),f_{2}\left( x \right) \right), wheref_{1}\left( x \right)=x_{1},f_{2}\left( x,g \right)=g\left( x \right).\left( 1-\sqrt{\frac{f_{1}}{g\left( x \right)}} \right),$$

$and,g\left( x \right)=1+\frac{9}{n-1}.\sum_{i=2}^{n} x_{i},$ Constraints: $0\leq x_{i}\leq1,n=30, i=1,2,\ldots,30$

A.2 ZDT 2 with non-convex POF:

$$F=\left( f_{1}\left( x \right),f_{2}\left( x \right) \right), wheref_{1}\left( x \right)=x_{1},f_{2}\left( x,g \right)=g\left( x \right).\left( 1-\left( \sqrt{\frac{f_{1}}{g\left( x \right)}} \right)^{2} \right),$$

$and,g\left( x \right)=1+\frac{9}{n-1}.\sum_{i=2}^{n} x_{i},$ Constraints $0\leq x_{i}\leq1,n=30, i=1,2,\ldots,30$

A.3 ZDT 3 with convex and discrete POF:

$$F=\left( f_{1}\left( x \right),f_{2}\left( x \right) \right), wheref_{1}\left( x \right)=x_{1},f_{2}\left( x,g \right)=g\left( x \right).\left( 1-\sqrt{\frac{f_{1}}{g\left( x \right)}}-\frac{f_{1}}{g\left( x \right)}.sin(10\pi f_{1}) \right),$$

$and,g\left( x \right)=1+\frac{9}{n-1}.\sum_{i=2}^{n} x_{i},$Constraints: $0\leq x_{i}\leq1,n=30, i=1,2,\ldots,30$

A.4 ZDT 4 with non-convex POF:

$$F=\left( f_{1}\left( x \right),f_{2}\left( x \right) \right), wheref_{1}\left( x \right)=x_{1},f_{2}\left( x,g \right)=g\left( x \right).\left( 1-\sqrt{\frac{f_{1}}{g\left( x \right)}} \right),$$

$$and,g\left( x \right)=1+10.\left( n-1 \right)+\sum_{i=2}^{n} \left( x_{i}^{2}-10cos(4\pi x_{i}) \right),$$

Constraints: $0\leq x_{1}\leq1,-5\leq x_{i}\leq5,n=10, i=1,2,\ldots,10$

A.5 ZDT 6 with non-uniformly and non-convex POF:

$$F=\left( f_{1}\left( x \right),f_{2}\left( x \right) \right), wheref_{1}\left( x \right)=1-\exp\left( 4x_{1} \right).{sin}^{6}(6\pi x_{1}),f_{2}\left( x,g \right)=g\left( x \right).\left( 1-\left( \frac{f_{1}}{g\left( x \right)} \right)^{2} \right),$$

$and,g\left( x \right)=1+\frac{9}{n-1}.\left[ \frac{\sum_{i=2}^{n} x_{i}}{9} \right]^{0.25},$Constraints: $0\leq x_{i}\leq1,n=10, i=1,2,\ldots,10$

**Appendix B: Unconstrained multi-objective DTLZ with 2-Dim [63] test problems utilized in this work.**

B.1 DTLZ 1 with linear POF:

$$F=\left( f_{1}\left( x \right),f_{2}\left( x \right) \right), wheref_{1}\left( x \right)=\frac{1}{2}x_{1}\left( 1+g\left( x \right) \right),f_{2}\left( x \right)=\frac{1}{2}(1-x_{1})(1+g(x))$$

$$and,g\left( x \right)=100\left[ 1+\sum_{i=3}^{n} {(x_{i}-0.5)}^{2}-cos(20\pi(x_{i}-0.5)) \right],$$

Constraints: $0\leq x_{i}\leq1,n=12, i=1,2,\ldots,12$

B.2 DTLZ 2 with concave POF:

$$F=\left( f_{1}\left( x \right),f_{2}\left( x \right) \right), wheref_{1}\left( x \right)=cos\left( \frac{\pi}{2}x_{1} \right)\left( 1+g\left( x \right) \right),f_{2}\left( x \right)=sin\left( \frac{\pi}{2}x_{1} \right)\left( 1+g\left( x \right) \right)$$

$and,g\left( x \right)=\sum_{i=3}^{n} {(x_{i}-0.5)}^{2},$Constraints: $0\leq x_{i}\leq1,n=12, i=1,2,\ldots,12$

B.3 DTLZ 3 with concave POF:

$$F=\left( f_{1}\left( x \right),f_{2}\left( x \right) \right), wheref_{1}\left( x \right)=cos\left( \frac{\pi}{2}x_{1} \right)\left( 1+g\left( x \right) \right),f_{2}\left( x \right)=sin\left( \frac{\pi}{2}x_{1} \right)\left( 1+g\left( x \right) \right)$$

$$and,g\left( x \right)=100\left[ 1+\sum_{i=3}^{n} {(x_{i}-0.5)}^{2}-cos(20\pi(x_{i}-0.5)) \right],$$

Constraints: $0\leq x_{i}\leq1,n=12, i=1,2,\ldots,12$

B.4 DTLZ 4 with concave POF:

$$F=\left( f_{1}\left( x \right),f_{2}\left( x \right) \right), wheref_{1}\left( x \right)=cos\left( \frac{\pi}{2}x_{1}^{\alpha} \right)\left( 1+g\left( x \right) \right),f_{2}\left( x \right)=sin\left( \frac{\pi}{2}x_{1}^{\alpha} \right)\left( 1+g\left( x \right) \right)$$

$and,g\left( x \right)=\sum_{i=3}^{n} {(x_{i}-0.5)}^{2},$Constraints: $0\leq x_{i}\leq1,n=12, \alpha=100, i=1,2,\ldots,12$

B.5 DTLZ 5with curve POF:

$$F=\left( f_{1}\left( x \right),f_{2}\left( x \right) \right), wheref_{1}\left( x \right)=cos\left( \frac{\pi}{2}\theta_{1} \right)\left( 1+g\left( x \right) \right),f_{2}\left( x \right)=sin\left( \frac{\pi}{2}\theta_{1} \right)\left( 1+g\left( x \right) \right)$$

$$and,\theta_{1}=x_{1}.\left( \frac{\pi}{2} \right),g\left( x \right)=\sum_{i=3}^{n} {(x_{i}-0.5)}^{2},$$

Constraints: $0\leq x_{i}\leq1,n=12, i=1,2,\ldots,12$

B.6 DTLZ 6with curve POF:

$$F=\left( f_{1}\left( x \right),f_{2}\left( x \right) \right), wheref_{1}\left( x \right)=cos\left( \frac{\pi}{2}\theta_{1} \right)\left( 1+g\left( x \right) \right),f_{2}\left( x \right)=sin\left( \frac{\pi}{2}\theta_{1} \right)\left( 1+g\left( x \right) \right)$$

$and,\theta_{1}=x_{1}.\left( \frac{\pi}{2} \right),g\left( x \right)=\sum_{i=3}^{n} {(x_{i})}^{0.1},$Constraints: $0\leq x_{i}\leq1,n=12, i=1,2,\ldots,12$

B.7 DTLZ 7 with disconnected POF:

$$F=\left( f_{1}\left( x \right),f_{2}\left( x \right) \right), wheref_{1}\left( x \right)=x_{1},f_{2}\left( x \right)=(1+g\left( x \right)).h(f_{1},f_{2},g(x))$$

$$and,g\left( x \right)=1+\frac{9}{22}\sum_{i=3}^{n} {(x}_{i}),h\left( f_{1},f_{2},g\left( x \right) \right)=2-\sum_{i=1}^{2} \left( \frac{f_{i}}{1+g}(1+sin(3\pi f_{i})) \right)$$

Constraints: $0\leq x_{i}\leq1,n=22, i=1,2,\ldots,12$

**Appendix C: Unconstrained multi-objective DTLZ with 3-Dim [63] test problems utilized in this work.**

C.1 DTLZ 1 with linear POF:

$$F=\left( f_{1}\left( x \right),f_{2}\left( x \right),f_{3}\left( x \right) \right), wheref_{1}\left( x \right)=\frac{1}{2}x_{1}x_{2}\left( 1+g\left( x \right) \right),f_{2}\left( x \right)=\frac{1}{2}x_{1}(1-x_{2})(1+g(x))$$

$$f_{3}\left( x \right)=\frac{1}{2}(1-x_{1})(1+g(x))and,g\left( x \right)=100\left[ 10+\sum_{i=3}^{n} {(x_{i}-0.5)}^{2}-cos(20\pi(x_{i}-0.5)) \right],$$

Constraints: $0\leq x_{i}\leq1,n=12, i=1,2,\ldots,12$

C.2 DTLZ 2 with concave POF:

$$F=\left( f_{1}\left( x \right),f_{2}\left( x \right),f_{3}\left( x \right) \right), wheref_{1}\left( x \right)=cos\left( \frac{\pi}{2}x_{1} \right)cos\left( \frac{\pi}{2}x_{2} \right)\left( 1+g\left( x \right) \right),$$

$$f_{2}\left( x \right)=cos\left( \frac{\pi}{2}x_{1} \right)sin\left( \frac{\pi}{2}x_{2} \right)\left( 1+g\left( x \right) \right),f_{3}\left( x \right)=sin\left( \frac{\pi}{2}x_{1} \right)\left( 1+g\left( x \right) \right)$$

$and,g\left( x \right)=\sum_{i=3}^{n} {(x_{i}-0.5)}^{2},$Constraints: $0\leq x_{i}\leq1,n=12, i=1,2,\ldots,12$

C.3 DTLZ 3 with concave POF:

$$F=\left( f_{1}\left( x \right),f_{2}\left( x \right),f_{3}\left( x \right) \right), wheref_{1}\left( x \right)=cos\left( \frac{\pi}{2}x_{1} \right)cos\left( \frac{\pi}{2}x_{2} \right)\left( 1+g\left( x \right) \right),$$

$$f_{2}\left( x \right)=cos\left( \frac{\pi}{2}x_{1} \right)sin\left( \frac{\pi}{2}x_{2} \right)\left( 1+g\left( x \right) \right),f_{3}\left( x \right)=sin\left( \frac{\pi}{2}x_{1} \right)\left( 1+g\left( x \right) \right)$$

$$and,g\left( x \right)=100\left[ 10+\sum_{i=3}^{n} {(x_{i}-0.5)}^{2}-cos(20\pi(x_{i}-0.5)) \right],$$

Constraints: $0\leq x_{i}\leq1,n=12, i=1,2,\ldots,12$

C.4 DTLZ 4 with concave POF:

$$F=\left( f_{1}\left( x \right),f_{2}\left( x \right),f_{3}\left( x \right) \right), wheref_{1}\left( x \right)=cos\left( \frac{\pi}{2}x_{1}^{\alpha} \right)cos\left( \frac{\pi}{2}x_{2}^{\alpha} \right)\left( 1+g\left( x \right) \right),$$

$$f_{2}\left( x \right)=cos\left( \frac{\pi}{2}x_{1}^{\alpha} \right)sin\left( \frac{\pi}{2}x_{2}^{\alpha} \right)\left( 1+g\left( x \right) \right),f_{3}\left( x \right)=sin\left( \frac{\pi}{2}x_{1}^{\alpha} \right)\left( 1+g\left( x \right) \right)$$

$and,g\left( x \right)=\sum_{i=3}^{n} {(x_{i}-0.5)}^{2},$Constraints: $0\leq x_{i}\leq1,n=12, \alpha=100, i=1,2,\ldots,12$

C.5 DTLZ 5 with curve POF:

$$F=\left( f_{1}\left( x \right),f_{2}\left( x \right),f_{3}\left( x \right) \right), wheref_{1}\left( x \right)=cos\left( \frac{\pi}{2}\theta_{1} \right)cos\left( \frac{\pi}{2}\theta_{2} \right)\left( 1+g\left( x \right) \right),$$

$$f_{2}\left( x \right)=cos\left( \frac{\pi}{2}\theta_{1} \right)sin\left( \frac{\pi}{2}\theta_{2} \right)\left( 1+g\left( x \right) \right),f_{3}\left( x \right)=sin\left( \frac{\pi}{2}\theta_{1} \right)\left( 1+g\left( x \right) \right)$$

$$and,\theta_{1}=x_{1}.\left( \frac{\pi}{2} \right),\theta_{2}=\frac{\pi}{4.\left( 1+g\left( x \right) \right)}.(1+2x_{2}.g(x)),g\left( x \right)=\sum_{i=3}^{n} {(x_{i}-0.5)}^{2},$$

Constraints: $0\leq x_{i}\leq1,n=12, i=1,2,\ldots,12$

C.6 DTLZ 6 with curve POF:

$$F=\left( f_{1}\left( x \right),f_{2}\left( x \right),f_{3}\left( x \right) \right), wheref_{1}\left( x \right)=cos\left( \frac{\pi}{2}\theta_{1} \right)cos\left( \frac{\pi}{2}\theta_{2} \right)\left( 1+g\left( x \right) \right),$$

$$f_{2}\left( x \right)=cos\left( \frac{\pi}{2}\theta_{1} \right)sin\left( \frac{\pi}{2}\theta_{2} \right)\left( 1+g\left( x \right) \right),f_{3}\left( x \right)=sin\left( \frac{\pi}{2}\theta_{1} \right)\left( 1+g\left( x \right) \right)$$

$$and,\theta_{1}=x_{1}.\left( \frac{\pi}{2} \right),\theta_{2}=\frac{\pi}{4.\left( 1+g\left( x \right) \right)}.(1+2x_{i}.g(x)),g\left( x \right)=\sum_{i=3}^{n} {(x_{i})}^{0.1},$$

Constraints: $0\leq x_{i}\leq1,n=12, i=1,2,\ldots,12$

C.7 DTLZ 7 with disconnected POF:

$$F=\left( f_{1}\left( x \right),f_{2}\left( x \right),f_{3}\left( x \right) \right), wheref_{1}\left( x \right)=x_{1},f_{2}\left( x \right)=x_{2},f_{3}\left( x \right)=(1+g\left( x \right)).h(f_{1},f_{2},g(x))$$

$$and,g\left( x \right)=1+\frac{9}{22}\sum_{i=3}^{n} {(x}_{i}),h\left( f_{1},f_{2},g\left( x \right) \right)=3-\sum_{i=1}^{2} \left( \frac{f_{i}}{1+g}(1+sin(3\pi f_{i})) \right)$$

Constraints: $0\leq x_{i}\leq1,n=22, i=1,2,\ldots,12$

**Appendix D: Constrained multi-objective test problems [64,65] utilized in this work.**

D.1 CONSTR with convex POF:

$$F=\left( f_{1}\left( x,y \right),f_{2}\left( x,y \right) \right), wheref_{1}\left( x,y \right)=x,f_{2}\left( x,y \right)=(1+y)/x,$$

Constraints: $0.1\leq x\leq1, 0\leq y\leq5,0\geq6-y-9x,0\geq1+y-9x$

D.2 TNK with dis-connected and convoluted POF:

$$Minimize F=\left( f_{1}\left( x,y \right),f_{2}\left( x,y \right) \right), wheref_{1}\left( x,y,z \right)=x,f_{2}\left( x,y,z \right)=y,$$

Constraints: $0\leq x,y\leq\pi,0\geq-x^{2}-y^{2}+1+0.1*cos\left( 16arctan\frac{x}{y} \right),\frac{1}{2}\geq\left( x-\frac{1}{2} \right)^{2}+\left( y-\frac{1}{2} \right)^{2},$

D.3 SRN with connected POF:

$$F=\left( f_{1}\left( x,y \right),f_{2}\left( x,y \right) \right), wheref_{1}\left( x,y \right)={(x-2)}^{2}+{(y-1)}^{2}+2,f_{2}\left( x,y \right)=9x-{(y-1)}^{2},$$

Constraints: $-20\leq x,y\leq20,0\geq x^{2}+y^{2}-225,0\geq x-3y+10$

D.4 OSY with discrete POF:

$$F=\left( f_{1}\left( x \right),f_{2}\left( x \right) \right), wheref_{1}\left( x \right)=-\left( 25\left( x_{1}-2 \right)^{2}+\left( x_{2}-2 \right)^{2} \right)+\left( x_{3}-1 \right)^{2}+\left( x_{4}-4 \right)^{2}+{(x_{5}-1)}^{2},$$

$$f_{2}\left( x \right)=x_{1}^{2}+x_{2}^{2}+x_{3}^{2}+x_{4}^{2}+x_{5}^{2}+x_{6}^{2},$$

Constraints: $0\leq x_{1},x_{2},x_{6}\leq10,1\leq x_{3},x_{5}\leq5,0\leq x_{4}\leq6,0\leq x_{1}+x_{2}-2,0\leq6-x_{1}-x_{2},$

$$0\leq2-x_{2}+x_{1},0\leq2-x_{1}+3x_{1},0\leq4-\left( x_{3}-3 \right)^{2}-x_{4},0\leq\left( x_{5}-3 \right)^{2}+x_{6}-4,$$

D.5 BNH with convex POF:

$$F=\left( f_{1}\left( x,y \right),f_{2}\left( x,y \right) \right), wheref_{1}\left( x,y \right)=4x^{2}+4y^{2},f_{2}\left( x,y \right)={(x-5)}^{2}+{(y-5)}^{2},$$

Constraints: $0\leq x\leq5, 0\leq y\leq3,0\geq\left( x-5 \right)^{2}+\left( y \right)^{2}-25,0\geq{-\left( x-8 \right)}^{2}+\left( y+3 \right)^{2}+7.7$

D.6 KITA with discrete concave POF:

$$Maximize F=\left( f_{1}\left( x,y \right),f_{2}\left( x,y \right) \right), wheref_{1}\left( x,y \right)=-x^{2}+y,f_{2}\left( x,y \right)=\left( \frac{1}{2} \right)x+y+1,$$

Constraints: $x,y\geq0,0\geq(1/6)x+y-(13/2),0\geq(1/2)x+y-(15/2),0\geq5x+y-30,$

**Appendix E: Constrained multi-objective engineering problems used in this work.**

E.1 Brushless DC Wheel Motor design problem (RWMOP1):

Brushless DC wheel motor design problem is a constrained multi-objective problem in the area of electrical machine design [66]. The objectives are in conflict, minimize the total weight (*f1*) and efficiency (*f2*) should be maximized and there are five design variables: stator diameter (*Ds*), magnetic induction in the air gap (*Be*), current density in the conductors (*δ*), magnetic induction in the teeth (*Bd*) and magnetic induction in the stator back iron (*Bcs*) as well as five constraints. The pareto optimal front have non-convex and connected in nature.

| $f_{1}\left( x \right)=, f_{2}\left( x \right)=Min$ |  |
| --- | --- |
| Where: $G\left( x \right)=$ |  |
| $\begin{matrix} 150mm\leq D_{s}\leq330mm,0.5T\leq B_{e}\leq0.76T,2A/mm^{2}\leq\varsigma\leq5A/mm^{2},0.9T\leq B_{d}\leq1.8T \\ 0.6T\leq B_{cs}\leq1.6T \end{matrix}$ |  |

E.2 Helical Spring design problem (RWMOP2):

The Helical Spring design mixed variables optimization problem first proposed by Kannan and Kramer a well-known problem in the mechanical engineering field [65], in which stress (*f1*) and volume (*f2*) of a Helical Spring problem should be minimized. As can be seen in the following equations, there are three design variables (*x1-x3*), spring coils (*x1: an integer variable*), wire diameter (*x2: a discrete variable*), and spring diameter (*x3*) as well as eight constraints. The pareto optimal front have convex and multi-connected in nature.

$$Pmax=1000;C=\frac{x3}{x2};K=\left( \frac{4*C-1}{4*C-4} \right)+\left( 0.615*\frac{x2}{x3} \right); G=11500000;Vmax=30;dmin=0.2;S=189000;dpm=6;lmax=14;dw=1.25;Pmax=1000;Dmax=3;P=300;C=\frac{x3}{x2};k=\frac{G*x2^{4}}{8*x1*\left( x3^{3} \right)};$$

$$dp=\frac{P}{k};K=\left( \frac{4*C-1}{4*C-4} \right)+\left( 0.615*\frac{x2}{x3} \right);$$

$$f1=\left( 0.25*pi^{2}*\left( x2^{2} \right)*x3*\left( x1+2 \right) \right); f2=\left( 8*K*Pmax*\frac{x3}{pi*\left( x2^{3} \right)} \right);$$

$$g\left( 1 \right)=-\left( lmax-\left( \frac{Pmax}{k} \right)-\left( 1.05*x2*\left( x1+2 \right) \right) \right);g\left( 2 \right)=-\left( x2-dmin \right);$$

$$g\left( 3 \right)=-\left( Dmax-\left( x2+x3 \right) \right);g\left( 4 \right)=-\left( C-3 \right);g\left( 5 \right)=-\left( dpm-dp \right);g\left( 6 \right)=-\left( \left( \frac{Pmax-P}{k} \right)-dw \right);g\left( 7 \right)=-\left( S-\left( 8*K*Pmax*\frac{x3}{pi*\left( x2^{3} \right)} \right) \right);g\left( 8 \right)=-\left( Vmax-\left( 0.25*pi^{2}*\left( x2^{2} \right)*x3*\left( x1+2 \right) \right) \right);$$

$$x2=[0.009 0.0095 0.0104 0.0118 0.0128 0.0132 0.014 0.015 0.0162 \ldots$$

$$0.0173 0.018 0.020 0.023 0.025 0.028 0.032 0.035 0.041 0.047 0.054\ldots$$

$$0.063 0.072 0.080 0.092 0.105 0.120 0.135 0.148 0.162 0.177 0.192 0.207\ldots$$

$$0.225 0.244 0.263 0.283 0.307 0.331 0.362 0.394 0.4375 0.500];$$

$$x1=[1 2 3\ldots30 31 32];0<x3\leq3;dim=3;$$

E.3 Two bar truss design problem (RWMOP3):

The 2-bar truss design problem is a well-known problem in the structural optimisation field, in which structural volume (*f1*) and maximum stresses (*f2*) exerted on bars are minimized without elastic failure due to 100 kN load. Single-objective optimization problem of 2-bar truss is founded in Kirsch (1981). Later reformulated by Deb as a multi-objective optimization problem [65]. As can be seen in the following equations, there are two design variables (*x1-x2*) related to cross section area of truss bar members as well as single constraint. The pareto optimal front have convex and connected in nature.

$$sigma_{AC}=\frac{20*sqrt\left( 16+x3^{2} \right)}{x1*x3};sigma_{BC}=\frac{80*sqrt\left( 1+x3^{2} \right)}{x2*x3};$$

$$f1=\left( x1*sqrt\left( 16+x3^{2} \right) \right)+\left( x2*sqrt\left( 1+x3^{2} \right) \right);f2=\max\left( sigma_{AC},sigma_{BC} \right);$$

$$g\left( 1 \right)=\max\left( sigma_{AC},sigma_{BC} \right)-100000;dim=3;0<x1,x2\leq0.01; 1<x3\leq3$$

E.4 Welded beam design problem (RWMOP4):

The welded beam design problem first proposed by Deb[67]. The fabrication cost (*f1*) and deflection of the beam (*f2*) of a welded beam should be minimized in this problem. There are four design variables: the thickness of the weld (*x1*), the length of the clamped bar (*x2*), the height of the bar (*x3*) and the thickness of the bar (*x4*) as well as four constraints. The pareto optimal front have convex and connected in nature.

| $f_{1}\left( x \right)=1.10471*x(1)^2*x(2)+0.04811*x(3)*x(4)*(14.0+x(2))$ |  |
| --- | --- |
| $f_{2}\left( x \right)=65856000/(30*10^6*x(4)*x(3)^3)$ |  |

$g_{1}\left( x \right)=tau-13600$, $g_{2}\left( x \right)=sigma-30000$, $g_{3}\left( x \right)=x(1)-x(4)$, $g_{4}\left( x \right)=6000-P$

$0.125\leq x_{1}\leq5,0.1\leq x_{2}\leq10,0.1\leq x_{3}\leq10,0.125\leq x_{4}\leq5$Where$Q=6000*\left( 14+\frac{x\left( 2 \right)}{2} \right);$

$D=sqrt\left( \frac{x\left( 2 \right)^{2}}{4}+\frac{\left( x\left( 1 \right)+x\left( 3 \right) \right)^{2}}{4} \right)$, $J=2*\left( x\left( 1 \right)*x\left( 2 \right)*sqrt\left( 2 \right)*\left( \frac{x\left( 2 \right)^{2}}{12}+\frac{\left( x\left( 1 \right)+x\left( 3 \right) \right)^{2}}{4} \right) \right)$

$alpha=\frac{6000}{sqrt\left( 2 \right)*x\left( 1 \right)*x\left( 2 \right)}$, $beta=Q*\frac{D}{J}$, $P=tmpf*sqrt\left( x\left( 3 \right)^{2}*\frac{x\left( 4 \right)^{6}}{36} \right)*\left( 1-x\left( 3 \right)*\frac{sqrt\left( \frac{30}{48} \right)}{28} \right)$

$tau=sqrt\left( alpha^{2}+2*alpha*beta*\frac{x\left( 2 \right)}{2*D}+beta^{2} \right)$, $sigma=\frac{504000}{x\left( 4 \right)*x\left( 3 \right)^{2}}$, $tmpf=4.013*\frac{30*{10}^{6}}{196}$

E.5 Disk Brake Design Problem (RWMOP5):

The disk brake design problem has mixed constraints and was proposed by Osyczka and Kundu[68]. The objectives to be minimized are: stopping time (*f1*) and mass of a brake (*f2*) of a disk brake. As can be seen in following equations, there are four design variables: the inner radius of the disk (*x1*), the outer radius of the disk (*x2*), the engaging force (*x3*), and the number of friction surfaces (x4) as well as five constraints. The pareto optimal front have convex and connected in nature.

$$f1=4.9*\left( {10}^{-5} \right)*\left( x2^{2}-x1^{2} \right)*\left( x4-1 \right);f2=\frac{9.82*\left( {10}^{6} \right)*\left( x2^{2}-x1^{2} \right)}{\left( x2^{3}-x1^{3} \right)*x4*x3};$$

$$g\left( 1 \right)=20+x1-x2;g\left( 2 \right)=2.5*\left( x4+1 \right)-30;g\left( 3 \right)=\frac{x3}{3.14*\left( x2^{2}-x1^{2} \right)^{2}}-0.4;$$

$$g\left( 4 \right)=\frac{2.22*{10}^{-3}*x3*\left( x2^{3}-x1^{3} \right)}{\left( x2^{2}-x1^{2} \right)^{2}}-1;g\left( 5 \right)=900-\frac{2.66*{10}^{-2}*x3*x4*\left( x2^{3}-x1^{3} \right)}{\left( x2^{2}-x1^{2} \right)};$$

$$dim=4;55<x1\leq80;75<x2\leq110; 1000<x3\leq3000; 2<x4\leq20;$$
